# Deciphering the molecular mechanism responsible for GCaMP6m's Ca^2+^-dependent change in fluorescence

**DOI:** 10.1371/journal.pone.0170934

**Published:** 2017-02-09

**Authors:** Lauren M. Barnett, Thomas E. Hughes, Mikhail Drobizhev

**Affiliations:** Department of Cell Biology and Neuroscience, Montana State University, Bozeman, Montana, United States; Russian Academy of Medical Sciences, RUSSIAN FEDERATION

## Abstract

The goal of this work is to determine how GCaMP6m’s fluorescence is altered in response to Ca^2+^-binding. Our detailed spectroscopic study reveals the simplest explanation for how GCaMP6m changes fluorescence in response to Ca^2+^ is with a four-state model, in which a Ca^2+^-dependent change of the chromophore protonation state, due to a shift in pK_a_, is the predominant factor. The pK_a_ shift is quantitatively explained by a change in electrostatic potential around the chromophore due to the conformational changes that occur in the protein when calmodulin binds Ca^2+^ and interacts with the M13 peptide. The absolute pK_a_ values for the Ca^2+^-free and Ca^2+^-saturated states of GCaMP6m are critical to its high signal-to-noise ratio. This mechanism has important implications for further improvements to GCaMP6m and potentially for other similarly designed biosensors.

## Introduction

Genetically-encoded Ca^2+^ sensors based on a single fluorescent protein (i.e. non-FRET based) are important imaging tools in neuroscience. The newest generation GCaMP6 sensors (GCaMP6s, GCaMP6m, and GCaMP6f, [1]) are bright and respond to transient increases in Ca^2+^ within the cell with such large changes in fluorescence that they can be used to image neuronal activity in awake, behaving animals, throughout large regions of the brain [2–5] and in entire brains [6,7].

**Fig 1:**
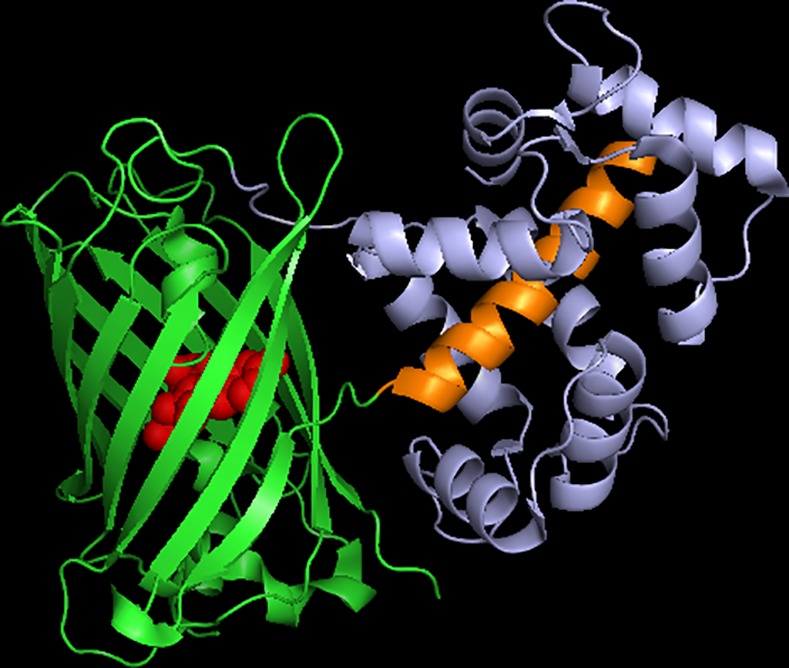
Schematic of Ca^2+^-saturated GCaMP6m structure. M13 domain (orange); circularly-permuted GFP domain (green); calmodulin domain (purple). Made using 3WLD.pdb [9].

The GCaMP sensors are constructed with circularly-permuted GFPs [8] in which the original N- and C-termini of the enhanced green fluorescent protein (EGFP) are linked together with a short, 6 amino acid peptide, and new termini are created in the middle of the 7th β-strand of the 11-stranded β-barrel, creating an opening in the side of the barrel directly adjacent to the phenolate oxygen in the chromophore. A portion of the Ca^2+^ binding protein calmodulin, and its target peptide the M13 domain, are individually fused to the new C- and N-termini of the circularly-permuted GFP, respectively (Fig 1). The fusion of these proteins at this very sensitive location couples the Ca^2+^-dependent calmodulin binding of M13 to large changes in the fluorescence of the circularly-permuted GFP.

The GCaMPs have been extensively studied and iteratively improved upon. More is known about the GCaMP sensors than any other biosensor, but there is still no quantitative molecular model that explains how these Ca^2+^ sensors produce such large changes in fluorescence. There are, however, several hints in the literature. First, it is clear that the pK_a_ (pK_a_ = -lgK_a_, where K_a_ is the acid dissociation constant) of the chromophore in the Ca^2+^ sensor is different for the Ca^2+^-free and Ca^2+^-saturated states [8,10–15]. Second, amino acids at key positions close to the chromophore are critical to the function of the Ca^2+^ sensor [9,11,12,14–17]. Third, the protein conformation of the Ca^2+^-free state allows increased solvent access directly to the chromophore, via the circularly-permuted GFP opening in the side of the protein barrel, compared to the Ca^2+^-saturated state [9,11,14,18].

Ultimately, there are only three ways for GCaMP fluorescence to change as a function of Ca^2+^-binding: the extinction coefficient can change, the quantum efficiency of emission can change, or there can be rapid changes in the concentration of the fluorescent chromophore. Our goal was to determine which of these mechanisms is most important.

## Results

### The mechanism of GCaMP6m is more complicated than a simple "dim" to "bright" transition of the chromophore

**Fig 2:**
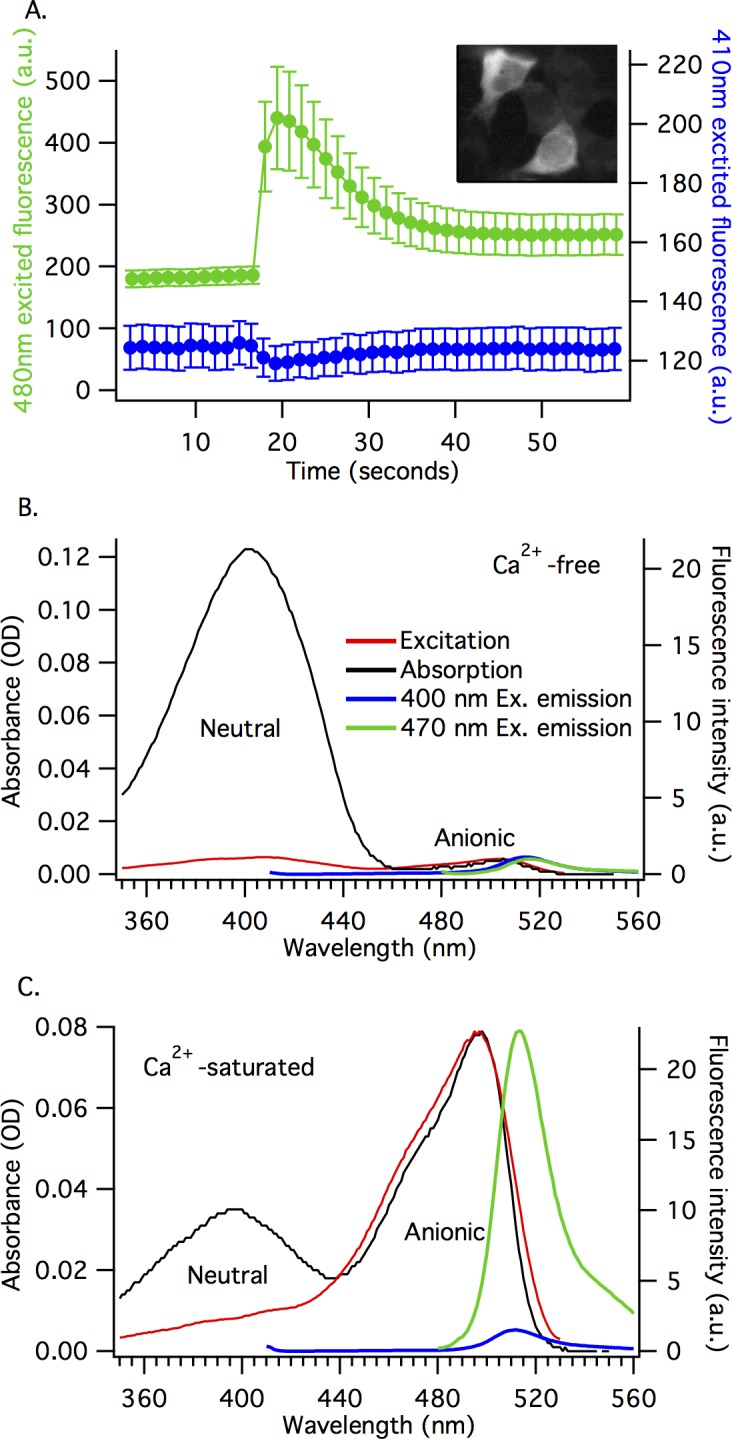
The Ca^2+^-dependent change in GCaMP6m fluorescence is more complicated than a chromophore transition from dim to bright. A) Transient changes in Ca^2+^ produce different responses in the 410 nm and 480 nm excited fluorescence of GCaMP6m. This graph shows GCaMP6m response to the release of intracellular Ca^2+^ stores in HEK293 cells (average of 12 cells, mean ± standard error). Green trace represents 480 nm excited fluorescence (left axis), Blue trace represents 410 nm excited fluorescence (right axis), fluorescence emission was collected at ≥ 515 nm. Muscarinic receptor (M1) activation was used to trigger the Ca^2+^ transient. Inset image: two HEK293 cells at peak value of 480 nm excited fluorescence. B and C) show that GCaMP6m exists in an equilibrium between at least two forms of the chromophore. Absorption spectra (black trace, left axis) and excitation spectra (red trace, right axis), and emission spectra for 400 nm excited fluorescence (blue trace, right axis) and 470 nm excited fluorescence (green trace, right axis) for purified GCaMP6m protein in 0 μM free Ca^2+^ buffer (Ca^2+^-free, B) and 39 μM free Ca^2+^ buffer (Ca^2+^-saturated, C). Fluorescence emission collected at 550 nm for excitation spectra.

GCaMP fluorescence is typically imaged with ~480 nm excitation (for one-photon excitation), observing the total emission peaking at ~510 nm [19–22]. Fig 2A illustrates that when Ca^2+^ levels inside the cell are low, there is little fluorescence, and when Ca^2+^ levels rise, so does the 480 nm excited fluorescence intensity (green trace, Fig 2A). The GCaMP6m response can also be observed using 410 nm excitation, but the fluorescence is weaker (with comparable excitation power and the same detection filter) and it decreases during the response to Ca^2+^ (blue trace, Fig 2A). This phenomenon was also observed for the earlier, GCaMP-like ratiometric pericam Ca^2+^ sensor [12], and indicates that the mechanism of GCaMP6m is more complicated than a simple chromophore transition from a dim state to a bright state.

To understand this behavior, we need to characterize the photophysical properties of GCaMP6m. The absorption spectra for purified GCaMP6m protein are different in Ca^2+^-free and Ca^2+^-saturated states (black traces, Fig 2B and 2C). For the Ca^2+^-free state, there is a dominant neutral form peak at 403 nm and a very small anionic form peak at 504 nm (Fig 2B). For Ca^2+^-saturated state, the absorption intensities redistribute such that the anionic form peak at 497 nm is dominant and the neutral form peak at 397 nm is smaller (Fig 2C). There is a notable blue-shift in the peak absorption of the anionic chromophore, from 504 nm in the Ca^2+^-free state to 497 nm in the Ca^2+^-saturated state (Fig 2B and 2C). This shift can be qualitatively explained by a change in the local electric field at the GCaMP6m chromophore due to Ca^2+^-dependent rearrangements of the chromophore surrounding [23].

The intensity distribution in the fluorescence excitation spectra is different from the absorption spectra. Whether the neutral or anionic form of the chromophore is excited, almost all of the GCaMP6m fluorescence emission comes from the anionic form of the chromophore (~515 nm), due to the excited state proton transfer (ESPT) that occurs from the electronically excited state of the neutral form of chromophore to a nearby proton accepting group [24–26]. The fluorescence excitation spectra (red traces, Fig 2B and 2C) are measured by detecting the fluorescence of the anionic form. By normalizing the excitation and absorption spectra with respect to the anionic form peak, it is clear that the excitation at the neutral form peak (~400 nm) produces a much weaker signal than expected from the neutral form absorption peak. This is true for both the Ca^2+^-free and Ca^2+^-saturated states of GCaMP6m.

If the quantum efficiency of the ESPT is much less than 1, the fluorescence efficiency of the indirectly-excited anionic form (via excitation of the neutral form) will be much smaller than that of the directly-excited anionic form and there will be a decrease of the excitation signal, compared to the corresponding absorption. The inefficient ESPT in GCaMP6m, compared to wild-type GFP, is most likely due to the T222 residue (T65 in EGFP) which breaks the essential proton transfer wire [24,27]. Without the proton transfer wire and with inefficient direct emission from the neutral form (blue trace, Fig 2), the relaxation of the neutral chromophore from the excited state to the ground state is dominated by a more efficient internal conversion [24]. By comparing the absorption and excitation spectra, the ratio of the quantum yields for the neutral and anionic forms in GCaMP6m can be roughly estimated: ~0.05:1 (neutral:anionic) for Ca^2+^-free, and ~0.055:1 for Ca^2+^-saturated states. These excitation spectra are consistent with previous measurements of GCaMP6m and other GCaMP genetically-encoded Ca^2+^ sensors [1,10,11,13,14,16,17,28].

The significant red-shift of the absorption peak of the anionic chromophore in both the Ca^2+^-free and Ca^2+^-saturated GCaMP6m (504 nm and 497 nm, respectively), relative to the parent EGFP (488 nm), can be explained by rearrangement of local hydrogen-bonding with the chromophore. Instead of three hydrogen bonds of the phenolate oxygen in EGFP (with T203, H148, and a water molecule) there are only two in GCaMP6m (with 2 water molecules) [9]. In GCaMP6m, the mutations T115V (T203V in EGFP) and H60E (H148E in EGFP) disrupt the hydrogen-bonding of these residues with the chromophore. Similar rearrangements in the hydrogen bond network of other GFP variants are known to result in the red shift of the anionic chromophore [23,24].

**Fig 3:**
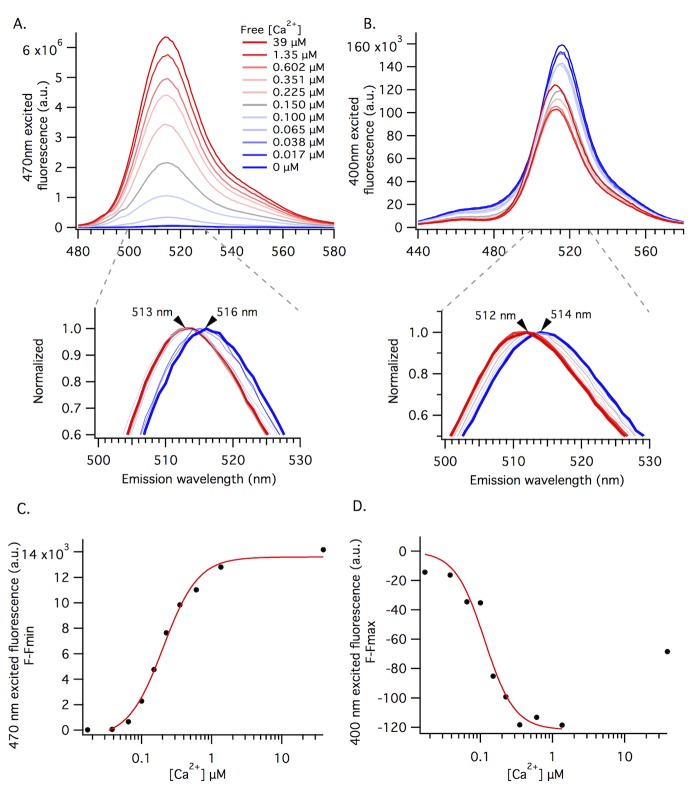
The GCaMP6m Ca^2+^-dependent spectra are consistent with four fluorescent states of the chromophore. A and B) illustrate the Ca^2+^ concentration dependent fluorescence of GCaMP6m. 470nm (top, A) and 400 nm (top, B) excited fluorescence spectra for purified protein at 11 different buffered free Ca^2+^ concentrations, ranging from 0 μM (dark blue trace) to 39 μM (dark red trace), at pH 7.2. The 400 nm excited spectra for 39 μM free Ca^2+^ sample is marked with black arrow (B). Extended lower graphs highlight Ca^2+^ concentration dependent λ_max_ with normalized 470 nm (A) and 400 nm (B) excited fluorescence (same spectra as top graphs). Arrows mark the apparent peak wavelength shift between 0 μM (dark blue trace) to 39 μM (dark red trace) buffered free Ca^2+^ concentrations. C and D) Peak fluorescence intensity values from spectra in 2A and 2B, respectively, plotted against buffered free Ca^2+^ concentration. F-F_min_ (y-axis, 2C) is used for 470 nm excited fluorescence because baseline fluorescence in the 0 μM free Ca^2+^ buffer is the minimum fluorescence at this excitation wavelength. Alternatively, F-F_max_ (y-axis, 2D) is used for 400 nm excited fluorescence because baseline fluorescence in the 0 μM free Ca^2+^ buffer is the maximum fluorescence at this excitation wavelength.

To examine the fluorescence behavior of the neutral and anionic forms of the chromophore upon binding Ca^2+^, we collected Ca^2+^-dependent fluorescence emission spectra with 470 nm (Fig 3A) and 400 nm (Fig 3B) excitation. The 470 nm excited fluorescence increases as a function of increasing Ca^2+^ concentration, whereas the 400 nm excited fluorescence decreases. The 400 nm excited fluorescence response is the opposite of the 470 nm excited response, but of qualitatively different magnitude. The Ca^2+^ concentration dependent behavior in the purified protein samples is similar to the fluorescent responses observed in our HEK293 cells experiments (Fig 2A).

Careful inspection reveals that there is a shift in the peak emission wavelength as a function of increasing Ca^2+^ concentrations in the spectra excited at 400 or 470 nm (Fig 3A and 3B). The peak emission of the anionic form blue-shifts 3 nm between the 0 and 39 μM buffered free Ca^2+^ samples when excited at 470 nm (from 516 to 513 nm, Fig 3A). The peak emission of the neutral form blue-shifts 2 nm between the 0 and 39 μM buffered free Ca^2+^ samples when excited at 400 nm (from 514 to 512 nm, Fig 3B). Normalized spectra in the expanded lower graphs highlight this shift for each Ca^2+^ titration. This reproducible shift (see S1 Fig for more data) is consistent with the shift in absorption of the anionic form, and inconsistent with a simple equilibrium between only two forms of the chromophore.

The peak fluorescence intensities for both the 470 and 400 nm excited emission spectra are plotted against buffered free Ca^2+^ concentration in Fig 3C and 3D, respectively. For the 470 nm excitation plot, the red line represents the best fit of the data to the Hill equation (Fig 3C), which illustrates the apparent Ca^2+^ affinity and cooperativity of GCaMP6m. The apparent K_d_ and Hill coefficient values (K_d_ = 164 ± 31 nM and n = 2.43 ± 0.44) are consistent with the previously reported values [1].

For the 400 nm excitation (Fig 3D), the red line is only a qualitative illustration of the decreasing fluorescence trend. The 400 nm excited fluorescence signal cannot be accurately fit with the Hill equation because it includes the emission of more than one form of the chromophore. One is the neutral form, peaking near 400 nm, and the other is the anionic form, peaking near 500 nm but with a long absorption tail extending near 400 nm. The Ca^2+^-saturated spectrum in Fig 3B (39 μM free Ca^2+^ buffer, red trace marked with black arrow) and the corresponding peak intensity in Fig 3D (final data point), illustrate this confounded measurement via an unexpected increase in 400 nm excited fluorescence. The relatively small fraction of the anionic form excited through the spectral tail overwhelms the response of the excited neutral form because it has a much larger fluorescence quantum yield.

### There are four different Ca^2+^-dependent states of GCaMP6m

**Fig 4:**
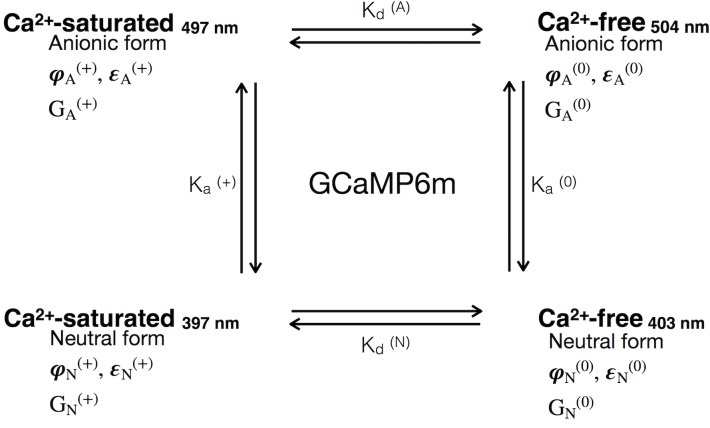
A four state model requires the quantitative characterization of each of the four distinct Ca^2+^-dependent states of GCaMP6m. This model illustrates a simple four state equilibrium that includes all possible combinations of the Ca^2+^-saturated and Ca^2+^-free states of the sensor protein, as well as anionic and neutral forms of the chromophore. Indices: Anionic form (A); Neutral (N); Ca^2+^-free (0); Ca^2+^-saturated (+); Quantum yield (φ); extinction coefficient (ε); Gibbs free energy of the protein (G); dissociation constant for calmodulin in GCaMP6m with anionic chromophore (K_d_^(A)^); dissociation for calmodulin in GCaMP6m with neutral chromophore (K_d_^(N)^); Acid dissociation constant for chromophore in Ca^2+^-saturated state of GCaMP6m (K_a_^(+)^); Acid dissociation constant for chromophore in Ca^2+^-free state of GCaMP6m (K_a_^(0)^).

A four-state model can explain the observed results. This model includes a Ca^2+^-free neutral form, a Ca^2+^-free anionic form, a Ca^2+^-saturated neutral form, and a Ca^2+^-saturated anionic form (Fig 4). Here, we assume that each state has a unique combination of photophysical parameters, including extinction coefficient, quantum yield and two-photon cross-section, and they are connected through equilibrium constants (K_d_ and K_a_). Each GCaMP6m state is also characterized by a unique electrostatic interaction energy of the chromophore with the surrounding protein (part of Gibbs free energy, G).

**Fig 5:**
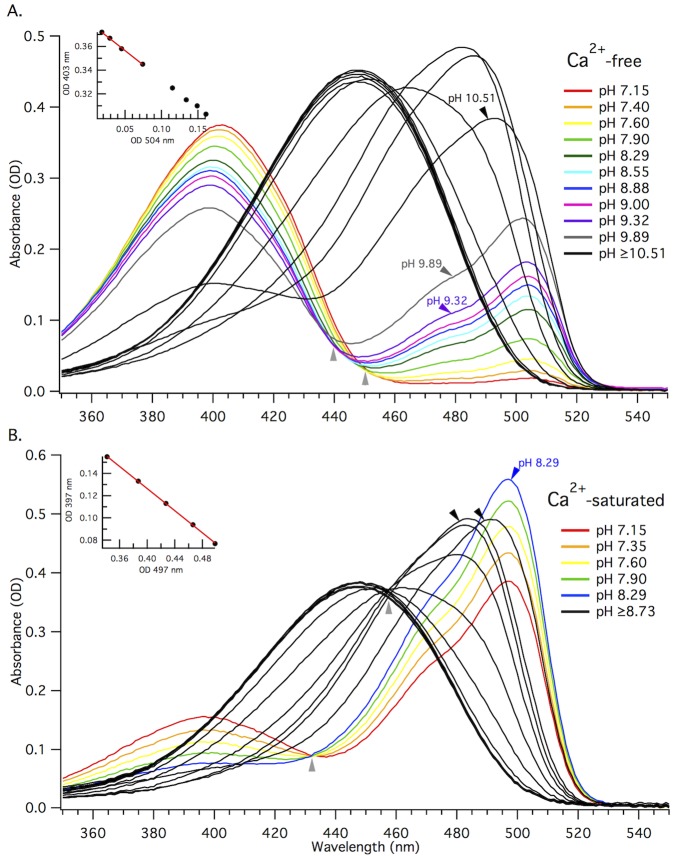
Alkaline titration makes it possible to measure the extinction coefficient of two different forms in the same sample. A and B) Absorption spectra for alkaline titration of purified GCaMP6m protein in 0 μM free Ca^2+^ buffer (Ca^2+^-free, A) or 39 μM free Ca^2+^ buffer (Ca^2+^-saturated, B). Individual traces represent absorption spectra at different pH values. The final two colored traces and the first black trace for Ca^2+^-free sample (A) are marked with arrows. The last colored trace and first two black traces for Ca^2+^-saturated sample are marked with arrows (B). This boundary marks a significant pH dependent change in the shape of the absorption peak near 500 nm and the last spectrum where we see linear intercorrelation of the change in OD for the two peaks. Light grey arrows below traces mark the isosbestic points. The peak value at 447 nm (black trace), gives the total concentration of chromophore. Inset plots: Absorbance values for 403 nm versus 504 nm taken from the first 8 colored traces (from pH 7.15 to 9) of the absorption spectra for Ca^2+^-free GCaMP6m (A), and for 397 nm versus 497 nm taken from all of the colored traces (from pH 7.15 to 8.29) for Ca^2+^-saturated GCaMP6m (B). These plots illustrate the change in OD for the neutral form (397 nm or 403 nm) relative to the anionic (497 nm or 504 nm) form for the respective pH ranges. The red linear fit of the first 4 data points (from pH 7.15 to 7.90) for Ca^2+^-free (A) and all 5 data points for Ca^2+^-saturated (B) in the change in OD plots gives us the ratio of the extinction coefficients for the neutral and anionic forms in the given sample.

With at least two forms of the chromophore in equilibria for a given sample, it is difficult to accurately measure the extinction coefficients for each form. The traditional 1-step alkaline denaturation of the entire sample is insufficient to resolve the concentrations of the individual chromophore species. Alkaline titration, however, makes it possible to measure both the ratio of the concentrations of two different forms of the chromophore and the total concentration of chromophore in the sample. Fig 5 illustrates the changes in absorption spectrum for the Ca^2+^-free (5A) and Ca^2+^-saturated (5B) purified protein samples as they are titrated from neutral to more alkaline pH.

For the Ca^2+^-free state, with increasing pH, the neutral form absorption peak decreases and the anionic form peak increases (Fig 5A), as expected. At pH values larger than 10.5, the anionic form peak shifts to shorter wavelengths (485 nm) and another peak belonging to the anionic chromophore of the denatured protein (447 nm, [29]) starts to grow, becoming the only peak at pH > 11.

There appears to be two isosbestic points, or a "smearing" of the spectra, for Ca^2+^-free state. The first point is at 450 nm and corresponds to absorption spectra between pH 7.15 and 7.90 (grey arrow, Fig 5A). The second isosbestic point is shifted to shorter wavelengths, at 440 nm, corresponding to absorption spectra between pH 8.29 and 9.32 (grey arrow, Fig 5A). The shift between the two isosbestic points is due to the emergence of an additional, shorter-wavelength absorbing anionic form (peaking at ~485 nm), that is not the denatured form (447 nm). This blue-shifted anionic form can be tentatively explained by the titration of a nearby acidic residue in GCaMP6m, E134 (E222 in EGFP), close to the deprotonated chromophore. Analogous spectral shifts are seen in the red fluorescent proteins, mCherry and mStrawberry, where the peaks also blue-shift because of the titration of the corresponding E215 residue at more alkaline pH [30].

The correlation between the optical densities of the two main absorption peaks in Ca^2+^-free GCaMP6m (Fig 5A, inset) maintains a consistent linear relationship (see methods for more details) between pH 7.15 and 9.89, before the shorter-wavelength peak starts to dominate. The slope of the linear fit for the first four data points is used to obtain the ratio of the two extinction coefficients for the neutral and anionic chromophore species. These points correspond to the spectra in the titration range of the first isosbestic point, before the emergence of a new form. The individual extinction coefficients for the Ca^2+^-free neutral and anionic chromophores are presented in Table 1.

**Table 1:** Photophysical parameters for GCaMP6m.

	ε_max_ M^-1^cm^-1^		φ		σ_2_ GM		*n*		pK_a_
	(λ_abs_, nm)		(λ_fluor_, nm)		(λ_abs_, nm)				
	Neutral	Anionic	Neutral	Anionic	Neutral	Anionic	Neutral	Anionic	
**Ca**^**2+**^**-free**	37,300	73,600	0.041	0.56	140	54	0.97	0.03	8.01,10.08
	(403)	(514)	(514)	(516)	(790)	(930)			*8*.68*
**Ca**^**2+**^**-saturated**	37,700	83,100	0.048	0.63	---	50	0.49	0.51	7.10
	(397)	(497)	(512)	(513)		(930)			*6*.90*
				*0*.61*					

Extinction coefficient (ε); quantum yield (*φ*); two-photon cross-section (*σ*_*2*_); relative concentration of chromophore (*n*), previously reported values for GCaMP6m are in italics.

*[1]

For the Ca^2+^-saturated state, with increasing pH, there is a gradual transition from the neutral form into the anionic form (Fig 5B). This transition is illustrated by a single, clean isosbestic point at 432 nm, corresponding to the absorbance spectra between pH 7.15 and 8.29. A clean isosbestic point indicates the presence of only two forms in this pH range. Similar to the Ca^2+^-free state, at pH ≥ 8.7, the anionic absorption peak significantly blue-shifts (~485 nm) most likely due to the titration of E134, before the protein is denatured (447 nm). Another isosbestic point at 455 nm corresponds to this second transition.

The inset graph (Fig 5B) illustrates the linear relationship between the optical densities of the neutral and anionic chromophore in Ca^2+^-saturated GCaMP6m within the titration range of the first isosbestic point (pH 7.15 and 8.29), from which we extracted the ratio of the two individual extinction coefficients. The individual Ca^2+^-saturated extinction coefficients and fluorescence quantum yields measured for each of the four distinct fluorescent forms in GCaMP6m are listed in Table 1.

The extinction coefficient of the neutral form of the chromophore is almost identical for the Ca^2+^-free and Ca^2+^-saturated states (Table 1). The extinction coefficient of the anionic form of the Ca^2+^-saturated state is 14% larger than that of the Ca^2+^-free state (Table 1). There are also very small changes in the quantum yields for the neutral form and the anionic form upon binding Ca^2+^ (Table 1).

The measurement of ΔF/F_0_ for GCaMP6m is wavelength dependent because the anionic chromophore absorption blue shifts when GCaMP6m binds Ca^2+^ (Fig 5A and 5B). The maximum relative change of fluorescence of anionic form between Ca^2+^-free and Ca^2+^-saturated states occurs when exciting near 470 nm, ΔF/F_0_ = 30 (see S2 Fig). At 470 nm, there is a 25% increase in extinction coefficient of the anionic form and 13% increase in quantum yield upon binding Ca^2+^, which cannot explain ΔF/F_0_ = 30. The predominant factor responsible for the large Ca^2+^-dependent change in 470 nm excited GCaMP6m fluorescence is most likely a dramatic change in the relative concentration of the neutral and anionic forms of the chromophore.

### There is a Ca^2+^-dependent change in pK_a_ for the GCaMP6m chromophore

**Fig 6:**
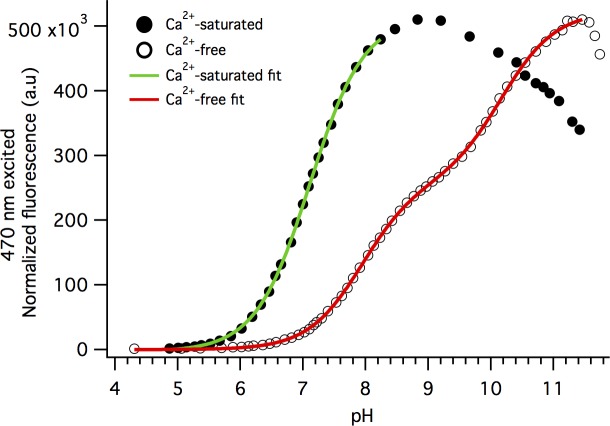
GCaMP6m has a single pKa for the Ca^2+^-saturated state and two pK_a_'s for the Ca^2+^-free state. Normalized 470 nm excited fluorescence intensity at 515 nm plotted against pH for Ca^2+^-saturated (solid circles) and Ca^2+^-free (open circles) states. A single-binding site model fit (green trace) was used for the Ca^2+^-saturated state and includes data points for one titration event from pH 4.3 to 8.39 with a pK_a_ of 7.10 ± 0.01. A two-binding site model fit (red trace) was used for the Ca^2+^-free state and includes data points for two titration events from pH 4.3 to 10.5, the first with a pK_a_ of 8.01 ± 0.01, and the second with a pK_a_ of 10.08 ± 0.02.

The shift of the neutral—anionic equilibrium upon binding Ca^2+^ is driven by a change of the pK_a_ value of the GCaMP6m chromophore. To examine this shift quantitatively, we collected fluorescence emission from the anionic GCaMP6m chromophore excited at 470 nm as a function of pH, for the Ca^2+^-saturated and Ca^2+^-free states. Both the Ca^2+^-saturated and Ca^2+^-free states of GCaMP6m exhibit an increase in peak fluorescence as a function of increasing pH. For the Ca^2+^-saturated state, the fluorescence signal increases up to pH ~8.9 (solid circles, Fig 6). The last two data points in the Ca^2+^-saturated titration at pH 8.47 and 8.83, before fluorescence starts to decrease, do not correspond to the initial anionic form alone. The signals in this range include emission from the blue-shifted anionic form observed in the extinction coefficient absorption measurements (first two black traces, Fig 5B). To get the apparent pK_a_ for the chromophore, we only used the data for pH from 4 to 8.39 (colored traces, Fig 5B; green fit trace, Fig 6), giving us a pK_a_ of 7.10 ± 01 for Ca^2+^-saturated GCaMP6m. This pK_a_ is slightly higher than previously reported value [1].

There are two distinct titration events that show up in the pH titration curve for the Ca^2+^-free state (open circles, Fig 6), which requires a two-binding site model. After pH ~11.5, the protein begins to denature and the 470 nm excited fluorescence decreases because the chromophore in denatured state does not fluoresce. The apparent pK_a_ of the Ca^2+^-free GCaMP6m chromophore in the first range is 8.01 ± 0.01. The second titration at the larger pH values most likely corresponds to the deprotonation of E134, and the apparent pK_a_ for this range is 10.08 ± 0.02. If all the data are fitted with a one-binding site model curve, the pK_a_ will be close to 9.0, similar to what has been previously reported [1]. The large change in pK_a_ between the first titration curve of Ca^2+^-free and Ca^2+^-saturated states (8.01 to 7.10) results in an increase in the concentration of the anionic chromophore at neutral pH, and consequently, a significant increase of the fluorescence signal excited at 470 nm.

The pH titration fluorescence measurements are wavelength dependent. Using a more red shifted excitation wavelength for the pH-dependent fluorescence measurements (488 nm versus 470 nm, for example) limits the resolution of the multiple chromophore species that show up as pH increases, making it more difficult to determine individual pK_a_ values.

### To better understand GCaMP6m, we need to understand the molecular mechanisms of the Ca^2+^-dependent change in pK_a_

The most significant factor in GCaMP6m's large change in fluorescence is a Ca^2+^-dependent change of pK_a_. In proteins, the pK_a_ depends on electrostatic potential energies of interactions between a titratable group, the GCaMP6m chromophore in this case, and the surrounding protein [31]. When the calmodulin domain in GCaMP6m binds Ca^2+^, there is a significant conformational change in the protein and several charged amino acid residues shift position relative to the chromophore. The displacement of these charged residues results in a change of potential at the chromophore and, consequently, pK_a_ of the chromophore. Determining the main amino acids contributing to this change will help to better understand the GCaMP sensors and could prove useful in future improvement of GCaMP6m.

The pK_a_ is related to the change of the free energy (ΔG) that occurs in the process of proton transfer from a titrating group to the surrounding water molecules [31]:

$$2.3RT∙{pK}_{a}^{\left(p\right)}=G_{s}^{\left(p\right)}(A^{-})-G_{s}^{\left(p\right)}(AH)+G_{s}^{\left(w\right)}(A^{-})-G_{s}^{\left(w\right)}(AH)+2.3RT∙{pK}_{a}^{\left(w\right)}$$ (1)

where pK_a_^(w)^ corresponds to pK_a_ of the chromophore in water (w), pK_a_^(p)^ corresponds to the pK_a_ of the chromophore surrounded by the protein barrel (p), and G_s_ is the solvation energy of either the anionic (A-) or neutral chromophore (AH). We only consider the difference between the pK_a_ values for the Ca^2+^-free (0) and Ca^2+^-saturated (+) states. Applying Eq 1 to these two different states and subtracting one from another to get the ΔpK_a_, we obtain,

$$\Delta {pK}_{a}={pK}_{a}^{\left(p,0\right)}-{pK}_{a}^{\left(p,+\right)}=\frac{\Delta \Delta G_{s}}{2.3RT}$$ (2)

where,

$$\Delta \Delta G_{s}={\Delta G}_{s}^{\left(p,0\right)}-{\Delta G}_{s}^{\left(p,+\right)}$$ (3)

and

$$\Delta G_{s}^{\left(p\right)}=G_{s}^{\left(p\right)}(A^{-})-G_{s}^{\left(p\right)}(AH)$$ (4)

Eq 3 describes the difference between the changes of free energy of the proton transfer reaction upon binding Ca^2+^.

It is essential to calculate the ΔΔG_s_ contributions from various amino acid residues in the chromophore environment to identify those that are most important for the Ca^2+^-dependent change in pK_a_. All of the free energy contributions from amino acids that do not move between the Ca^2+^-free and Ca^2+^-saturated states cancel in calculating ΔΔG_s_, therefore we only consider the ΔG_s_^(0)^ and the ΔG_s_^(+)^ contributions for amino acids with a Ca^2+^-dependent change of position in proximity to the chromophore. We examined charged amino acid residues inside the pocket close to the circularly-permuted GFP opening and occupying the surfaces of GFP-M13 linker in both the Ca^2+^-free and Ca^2+^-saturated states: the α3 helix in calmodulin for the Ca^2+^-saturated state, α4 and α5 helices of calmodulin in the Ca^2+^-free and Ca^2+^-saturated states, the linker between α4 and α5 of calmodulin in the Ca^2+^-free and Ca^2+^-saturated states, and the linker between β6 strand in GFP and α1 helix in calmodulin for the Ca^2+^-free state. The selection of residues belonging to the pocket is somewhat subjective, but we picked those that interact with the chromophore directly. Direct interaction was defined as an unobstructed (by any other domain of the protein) line of connection between the residue and the center of chromophore (the CB2 atom). These residues represent the "first shell" of the chromophore surrounding. We assume other charged amino acids in more distant "shells" are screened by bulk water and therefore not contributing to the total electrostatic potential of the chromophore environment.

To calculate the electrostatic potential energy, we apply the point charge model for the amino acid residues and a set of Mulliken charges on the chromophore atoms for both anionic and neutral forms [32,33]:

$$G=1390\Sigma_{i}\frac{Qq_{i}}{R_{i}}$$ (5)

where G is calculated in kJ/mol, Q is the charge on the residue (in units of elementary charge), q_i_ is the charge on the i-th atom of chromophore, and R_i_ is the distance between Q and q_i_ (in Angstrom). For all potential energy calculations, we used the GCaMP2 protein as a template because both the Ca^2+^-free and Ca^2+^-saturated GCaMP2 crystal structures are available. The coordinates of the atoms for the Ca^2+^-free monomeric form were taken from the 3EKJ pdf file [11] amended with a Ca^2+^-free calmodulin structure (1CFD pdb file [34]) by overlaying the two proteins (to the best coincidence) using UCSF Chimera software. For the Ca^2+^-saturated monomeric protein we used the 3EK4 pdb file [11]. The structures of circularly-permuted EGFP (3EVP.pdb [18]), and Ca^2+^-saturated monomeric GCaMP2-ΔRSET (3EVR.pdb [18]), GCaMP3-ΔRSET (4IK5.pdb [35]) and GCaMP6m (3WLD.pdb [9]) proteins were also used to obtain additional information on the position of water molecules and amino acids in GCaMP6m that were missing in the GCaMP2 structure.

To check our model, we first considered the effect of point mutations on ΔpK_a_. If a charged amino acid is removed from the structure, the ΔΔG and consequently ΔpK_a_ will change because of the eliminated electrostatic interaction. A single mutation D380Y in GCAMP3 protein was made to make the GCaMP5A protein [14]. This substitution results in a ΔpK_a_ = 1.93 for GCaMP5A, compared to the ΔpK_a_ = 1.43 for GCAMP3 [14]. In GCaMP3, the D380 residue is in the linker between the α4 and α5 helices of CaM, directly above the opening of GFP barrel. It is positioned approximately 31 Å from the center of the chromophore in the Ca^2+^-free state and moves closer, ~15 Å from the center of the chromophore, in the Ca^2+^-saturated state. The D380 residue belongs to the "first shell" of the chromophore surrounding. The calculated D380 ΔΔG contribution is ΔΔΔG = -73.0 kJ/mol, which corresponds to a change of ΔpK_a_ by -12.6. This means that the removal of the D380 charge with the D380Y mutation should result in an increase of ΔpK_a_ by 12.6 units. Although there is an increase in ΔpK_a_ between the two proteins, magnitude of the calculated difference is much larger than what is observed experimentally, ΔΔpK_a_ = 0.5 [14].

One explanation for this difference could be that we disregard the chromophore polarizability, i.e. the redistribution of electronic density that occurs when GCaMP3 binds Ca^2+^ and the negative charge of D380 moves closer to the chromophore. This charge—induced dipole interaction would reduce the interaction energy by, (1/2)α ∙[e/R^2^]^2^, where α is the polarizability of the chromophore in the ground state, and R is the distance between the center of chromophore and D380 side chain in the corresponding Ca^2+^-dependent state. Using the polarizability value for the GFP chromophore, α = 76 x 10^−24^ cm^3^ [36], and the distances described in the previous paragraph, we estimate the correction to ΔΔΔG < 1 kJ/mol (absolute value), which cannot explain the observed difference between our calculated ΔΔΔG value (73.0 kJ/mol) and what is expected from the experimentally measured ΔpK_a_ (ΔΔΔG = 2.9 kJ/mol).

**Fig 7:**
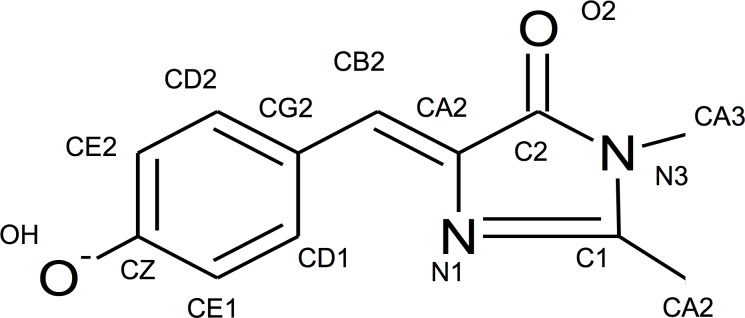
Atom numbering for the GCaMP chromophore.

Large discrepancies between experimental and calculated values of electrostatic interaction energies have been observed for similar systems in which a charged amino acid moves relative to a fluorescent probe (tryptophan residue) at the protein surface, if only protein charges are considered [37]. The discrepancies are entirely resolved if point charges from all water molecules within ~15 Å of the fluorescent probe are included, because of the opposing electrostatic field from the water molecules ordered by the protein charges (dielectric compensation) [37]. In circularly-permuted GFP, the opening in the β-barrel allows water molecules to access and hydrogen-bond with the chromophore, especially the phenolate moiety [11,18]. All of this considered, we make two important assumptions: one is that for part of the chromophore, the change of potential due to displacement of D380 is fully compensated by the dielectric response of nearby water molecules; and the second is that because the number of water molecules decreases towards the center of the protein barrel, the atoms with full compensation are most likely closer to the circularly-permuted opening. With these assumptions, we can make a series of calculations for the interaction energy (Eq 5) by sequentially setting the electric potential of each chromophore atom equal to zero, starting with a single atom, the phenolate oxygen closest to the circularly-permuted opening, and moving inward toward the imidazolinone ring, further from the circularly-permuted opening (Fig 7). The corresponding potential energies (Table 2) demonstrate that as more atoms experience an electric potential of zero, the calculated ΔΔpK_a_ value between GCaMP3 and GCaMP5A (due to the mutation D380Y) approaches the experimental value. The calculated ΔΔpK_a_ is consistent with the experimental ΔΔpK_a_ once the atom potentials from the phenolate oxygen to the CB2 atom are set to zero (i.e. considering only the electric potentials on all of the atoms of the imidazolinone ring).

**Table 2:** Calculated changes of ΔΔG and ΔpK_a_ for the transition from GCaMP3 to GCaMP5A in a model that assumes different sets of chromophore atoms interacting with D380 residue.

	Atoms included in calculation with electric potential of non-zero							Experimental Value*
	All	CZ to CA3	CE2 to CA3	CD2 to CA3	CG2 to CA3	CB2 to CA3	CA2 to CA3	
**ΔΔΔG (kJ/mol)**	-73.0	-30.8	-42.2	-16.3	-8.5	-13.5	-4.6	--
**ΔΔpK**_**a**_	-12.8	-5.4	-7.4	-2.9	-1.5	-2.4	-0.79	-0.5 ± 0.03

Each step of included atoms for the sequential calculations in our model (excluded atoms have electric potential equal to zero). Refer to Fig 7 for labelled illustration of the GCaMP chromophore.

*[14]

We checked this approach for the following GCaMP transitions: GCaMP3 to GCaMP5D, GCaMP5D to GCaMP5G, and GCaMP5G to GCaMP6m. In all of these cases, the calculations are consistent with the reported experimental values of ΔpK_a_ (Table 3). We assumed that K379S (GCaMP5G to GCaMP6m transition) is in the pocket only for the Ca^2+^-saturated state, R392G (GCaMP5G to GCaMP6m transition) is not in the pocket for both Ca^2+^-free and Ca^2+^-saturated states, and all other mutated charged residues (for all other transitions in Table 3) are in the pocket for both Ca^2+^-free and Ca^2+^-saturated states.

**Table 3:** The changes in ΔpK_a_ between recent GCaMP generations.

GCaMP Transitions	Relevant Charge Changing Mutations	Experimental ΔΔpK_a_*	Calculated ΔΔpK_a_
GCaMP3 to GCaMP5A	D380Y	0.50 ± 0.03	0.79
GCaMP3 to GCaMP5D	R303P	0.05 ± 0.06	0.02
GCaMP5D to GCaMP5G	D380Y	0.70 ± 0.08	0.79
GCaMP5G to GCaMP6m	K379S, T381R, R392G	-1.27 ± 0.06	-1.33

*[14]

This approach reveals critical charged amino acids that affect the ΔΔG and the corresponding Ca^2+^-dependent ΔpK_a_ for GCaMP2 and GCaMP6m, individually (Table 4). As before, we consider only those residues that fill the pocket around the circularly-permuted GFP opening and parts of calmodulin, and disregard those that do not move significantly upon Ca^2+^ binding (residues with ΔΔG < 1 kJ/mol). There are approximately 9–10 contributing charged amino acid residues for both proteins (Table 4). Their total effect on ΔpK_a_ agrees well with the experimental values (Table 4). The number of contributing residues and the magnitude of their contribution varies between the GCaMP generations. In GCaMP6m, E386 and E60 provide the largest effects (in absolute values), the rest contribute smaller amounts, in relatively equal proportions. In GCaMP2, the effect of E61 is very close to the total effect (ΔpK_a_ = 1.92 vs. 1.6, respectively) because the interactions of the rest of the residues almost cancel each other. This can explain why the point mutations E61K and E61G (GCaMP2 numbering) can reduce or completely eliminate the Ca^2+^-dependent change in fluorescence of GCaMP2 [18]. The important effects of amino acids E387, R377, D381, K380 (GCaMP2 numbering) on increasing or decreasing the Ca^2+^-dependent fluorescence response have also been demonstrated by point mutagenesis [11,18].

**Table 4:** Contributions to ΔΔG and ΔpK_a_ from important charged amino acids in GCaMP2 and GCaMP6m.

GCaMP2 amino acid	GCaMP6m amino acid	Position with respect to pocket		Contribution to ΔΔG (kJ/mol)		Contribution to ΔpK_a_	
		Ca^2+^-free	Ca^2+^-saturated	GCaMP2	GCaMP6m	GCaMP2	GCaMP6m
E61	E60	in	in	11.0	11.0	1.92	1.92
E357	E356	out	in	-6.8	-6.8	-1.19	-1.19
E387	E386	out	in	-16.3	-16.3	-2.86	-2.86
E386	E385	in	out	4.8	4.8	0.84	0.84
D381	Y380	in	in	-4.6	—	-0.81	—
D383	D382	out	in	-6.1	-6.1	-1.07	-1.07
D305	D304	in	out	6.8	6.8	1.19	1.19
R377	R376	out	in	5.4	5.4	0.95	0.95
K378	K377	out	in	6.1	6.1	1.07	1.07
K380	S379	out	in	9.0	—	1.58	—
T382	R381	in	in	—	1.4	—	0.246
Total ΔΔG (kJ/mol) or ΔpK_a_ (calculated):				9.3	6.3	1.6	1.1
Total ΔΔG (kJ/mol) or ΔpK_a_ (experimental):				—	—	1.7*	0.91

*[11]

### The two-photon absorption properties do not necessarily follow the same behavior as the one-photon absorption

**Fig 8:**
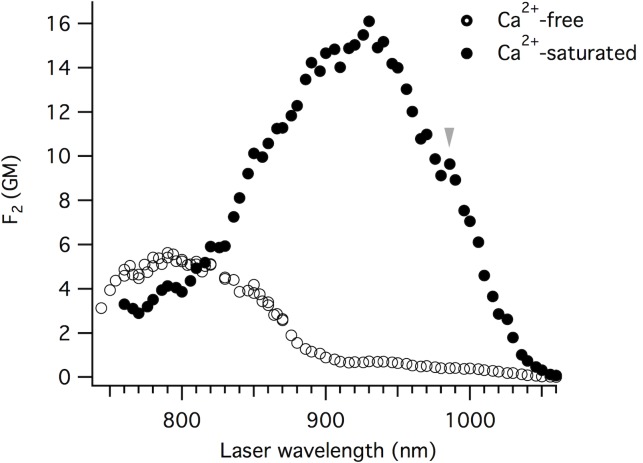
Two-photon excitation spectra for GCaMP6m. Spectra are presented as a linear combination of the two individual two-photon action cross-section spectra: F_2_ (λ) = σ_2_,_N_ (λ) φ_N_ ρ_N_ + σ_2_,_A_ (λ) φ_A_ ρ_A_, where σ_2_ (λ) is the wavelength-dependent two-photon excitation cross-section, φ is the fluorescence quantum yield, and ρ is the relative concentration of the chromophore species (neutral, N, or anionic, A). Ca^2+^-saturated (solid circles) and Ca^2+^-free (open circles).

The GCaMP6 sensors are important tools for imaging activity in living brains, much of which is done with two-photon excitation [2–7]. The two-photon absorption properties do not necessarily follow the same behavior as the one-photon properties [38]. Fig 8 illustrates the two-photon excitation spectra of GCaMP6m Ca^2+^-saturated and Ca^2+^-free states at neutral pH. In both states, the chromophore exists as a mixture of neutral (N) and anionic (A) forms (concentrations n_N_ and n_A_, respectively). Both forms of the chromophore are fluorescent within the same spectral region (green), have unique two-photon absorption spectra, and they contribute to the observed spectrum based on their relative concentrations, ρ_N_ = n_N_/(n_N_ + n_A_) and ρ_A_ = n_A_/(n_N_ + n_A_). Considering all of the above, the y-axis of Fig 8 represents a linear combination of the two individual two-photon action cross-section spectra: F_2_ (λ) = σ_2_,_N_ (λ) φ_N_ ρ_N_ + σ_2_,_A_ (λ) φ_A_ ρ_A_, where σ_2_(λ) is the wavelength-dependent two-photon excitation cross-section and φ is the fluorescence quantum yield.

For the Ca^2+^-saturated state, the spectrum is dominated by the anionic form, peaking at ~ 930 nm (black solid circles, Fig 8). This is because the neutral form has a significantly lower quantum yield (Table 1) and is in smaller relative concentration for the Ca^2+^-saturated state. To obtain F_2_(λ), we normalized the whole spectrum to the value of F_2_ = σ_2_,_A_(λ) φ_A_ ρ_A_ at 930 nm. Since the neutral form does not absorb at this wavelength, the latter was calculated as a product of independently measured σ_2_,_A_ (930 nm) = 50 GM, φ_A_ = 0.63 (Table 1), and ρ_A_ = 0.51 (obtained from linear absorption spectrum in Fig 2, and extinction coefficients ε_A_ and ε_N_ in Table 1). The two-photon excitation spectral peak for the anionic form (930 nm) is blue-shifted with respect to twice the wavelength of the one-photon absorption peak, grey arrow (994 nm) which can be explained by the enhancement of vibronic transition in two-photon absorption versus one-photon absorption, as a result of the unique Herzberg-Teller coupling effect [39,40].

In the Ca^2+^-free state, the neutral form dominates the two-photon excitation spectrum. The F_2_ spectrum for this state was normalized to the peak value at 800 nm, which was obtained from independently measured σ_2_,_N_ (800 nm) = 130 GM, φ_N_ = 0.041 (Table 1), and ρ_N_ = 0.97 (obtained similarly to ρ_A_ above). The neutral form two-photon excitation peak position (790 nm) is very close to twice the wavelength of the corresponding one-photon absorption peak, and the anionic form peak position is blue shifted with respect to twice the one-photon absorption peak.

We independently measured the two-photon absorption cross-section of the anionic form of the Ca^2+^-free state to be σ_2_(930 nm) = 54 GM. This value is very close to what would be expected from the F_2_(930 nm) value, obtained directly from the spectrum (Fig 8). This comparison is consistent with the fact that the equilibrium constant (ρ_A_ and ρ_N_ values) and quantum yields (φ_A_ and φ_N_ values) should be the same for one- and two-photon excited fluorescence. However, the two-photon cross-section for the neutral form (σ_2_(790 nm) = 140 GM) is 2.6 times larger than the anionic form (54 GM) contrary to the ratio of extinction coefficients, where the neutral form is 2 times smaller than the anionic form (Table 1).

Even though the Ca^2+^-free GCaMP6m two-photon fluorescence signal excited at 790 nm is much stronger compared to that of 930 nm (Fig 8), the excitation of the anionic form at 930 nm is still preferred for two-photon imaging. The anionic form produces a larger Ca^2+^-dependent change in fluorescence due to a much larger fluorescence quantum yield of the anionic form: ΔF_2,A_/ΔF_2,N_ = 5.6 (Fig 8).

The two-photon cross-sections for the neutral and anionic forms of GCaMP6m are significantly larger than the comparable non-circularly-permuted T203I EGFP mutant [41], even though they have similar one-photon absorption peak positions [23]. The σ_2,max_ for the anionic form of the T203I EGFP mutant is 19 GM [41], compared to 50 and 54 GM for Ca^2+^-saturated and Ca^2+^-free GCaMP6m, respectively. The increase in anionic form σ_2,max_ compared to T203I EGFP, is probably due to differences in the local surrounding of the chromophore, namely increased solvent access through the circularly-permuted opening and/or higher internal rotational freedom of the chromophore in both Ca^2+^-free and Ca^2+^-saturated states.

## Discussion

There is a significant difference in the quantum yield and extinction coefficients between the neutral and anionic form of the chromophore, but the photophysical parameters of the anionic form do not change much upon Ca^2+^-binding. When calmodulin binds Ca^2+^, it is the redistribution of the GCaMP6m population, from the neutral to the anionic form of the chromophore, that is responsible for the large change in ~470 nm excited fluorescence. The pK_a_ value of the chromophore strongly depends on specific amino acid positions in the chromophore environment. The change of the pK_a_ in our measurements, from 8.01 to 7.10, is consistent with the estimation of energies based on GCaMP2 and GCaMP6m crystal structures, as there are several essential charged amino acids that shift positions with respect to the chromophore when calmodulin binds Ca^2+^. We predict that E386, E60, E356, D304, D382, K377, R376, and E385 are the most important. The change in pK_a_ is important, but the absolute values of pK_a_ are important as well. Large signals will only be produced when the pK_a_ values of the Ca^2+^-free and Ca^2+^-saturated states straddle physiological pH. This relationship is illustrated in the comparison of the ΔF/F_0_ and pK_a_ values reported for various GCaMP/GCaMP-like genetically-encoded Ca^2+^ sensors [9–17,19,42] and other similarly designed circularly-permuted fluorescent protein biosensors [42–46].

The pK_a_ of Ca^2+^-saturated GCaMP6m (pK_a_ = 7.10) is very close to physiological pH. This means at pH 7.2, roughly 50% of the Ca^2+^-saturated GCaMP6m remains in the non-fluorescent (with ~470 nm for one-photon excitation or ~930 nm for two-photon excitation) neutral form of the chromophore. It also means that any small fluctuations of intracellular pH will affect the fluorescence signal of the Ca^2+^ sensor. At pH 7.2, a 0.2 pH unit fluctuation would result in a 17% change (increase or decrease, for alkaline or acidic pH fluctuations, respectively) in the Ca^2+^-saturated anionic chromophore fluorescence. If the pK_a_ of the Ca^2+^-saturated state was shifted to a more acidic pH, it would reduce GCaMP6m pH sensitivity and increase ΔF/F_0_. Altering the GCaMP6m design to include positive residues that move closer to chromophore when calmodulin binds Ca^2+^ would produce this acidic pK_a_ shift, increasing the ΔpK_a_ and the ΔF signal. As shown in Fig 6B, there is room to improve GCaMP6m ΔF by about ~70%. Rational or random mutagenesis of GCaMP6m combined with screening for specific pK_a_ properties [47], such as shifted pK_a_ values (i.e. more acidic values for Ca^2+^-saturated state) or increased ΔpK_a_ would provide more information regarding pK_a_ and ΔpK_a_ effects on Ca^2+^ sensor function, and could be beneficial for evolving GCaMP6m for increased ΔF without changing Ca^2+^-binding dynamic.

The four-state model we have applied for GCaMP6m could also be applied to understand the Ca^2+^-dependent fluorescence changes observed for other GCaMPs [9–17,19,28,42] and GCaMP-like biosensors [15,42–46,48–57]. iGluSnFR is a glutamate sensor based on a circularly-permuted GFP design similar to the GCaMPs [48]. The glutamate dependent pK_a_ shift reported for iGluSnFR is from 7.0 in the "free" state to 6.5 in the "bound" state, the relative concentrations of neutral and anionic forms change upon binding glutamate. FlinG2 and FlincG3 are cGMP sensors, also based on a GCaMP-like circularly-permuted GFP design, and report pK_a_ shifts of 7.48 (free) / 7.33 (bound) and 7.94 (free) / 7.53 (bound), respectively [43]. The FlincG biosensors also exhibit pH dependent changes in the ΔF signals, similar to what is seen for GCaMP6m. It is reasonable to consider that pK_a_ is important to how these sensors function, but to what extent is unclear. Applying a four-state model for these sensors, or any other circularly-permuted fluorescent protein biosensor [44–46,49–55,57], could reveal the predominant mechanism(s) responsible for their activity dependent changes in fluorescence and help improve future generations.

There are several clues in the literature that indicate a four-state model may also provide some insight into how ArcLight [58] and similar voltage sensors [59] function. ArcLight is constructed with a single, non-circularly-permuted, pH sensitive version of GFP, Super Ecliptic pHluorin [60] with an A227D mutation, fused to the intracellular C-terminal end of the voltage sensing domain of Ciona intestinalis voltage sensitive phosphatase (CiVSP) [58]. The Super Ecliptic pHluorin chromophore, like most GFP variants, exists in a protonation equilibrium. The neutral to anionic chromophore ratio is determined by its pK_a_ (~7.1) [60,61]. A change in the membrane potential of a cell expressing ArcLight triggers a conformational change in the voltage sensing domain of CiVSP, which translates to a decrease in the Super Ecliptic pHluorin A227D fluorescence. It is clear that the pH sensitivity of the fluorescent protein in ArcLight is essential for a voltage-dependent change in fluorescence [58,62]. It is also clear that a change in pH near the cell membrane during depolarization is not responsible for the ArcLight signal: when a pH sensitive protein is anchored at the membrane, without a voltage-sensitive protein, it does not respond to voltage changes [59,62]. Pado is a genetically-encoded voltage sensor consisting of a single, non-circularly-permuted super ecliptic pHluorin A227D fused to the intracellular C-terminal end of the voltage sensitive domain in a functional proton channel [59]. Pado is capable of monitoring changes in membrane potential and intracellular pH [59]. In altered extracellular pH experiments, at voltage steps large enough to open the proton conducting channel, Pado exhibits an increase in the magnitude of its 470 nm excited fluorescence response with an increase in pH, and a decrease in the magnitude of the 470 nm excited fluorescence response with a decrease in pH [59], much like GCaMP6m. One explanation for this data could be that Pado and ArcLight work like GCaMP6m and the reporting mechanism involves an essential voltage-dependent change in the pK_a_ of the pH-sensitive fluorescent protein, triggered by conformational changes in the CiVSP voltage sensing domain. Remarkably, there are very few measurements of the neutral form in these single GFP-based voltage sensors [63], or biophysical studies on the hyperpolarized and depolarized states [64], that might help substantiate this idea. Applying a four-state model to this family of sensors and exploring the importance of a voltage-dependent change in pK_a_ is certainly worth exploring, and will be a topic of our future investigation.

The remarkable redistribution of the GCaMP chromophore from neutral to anionic forms has two important consequences. First, with 470 nm excitation, the Ca^2+^ sensor is dark at low Ca^2+^ concentrations, and only fluorescent in active cells, producing an excellent signal-to-noise ratio in complex tissues like the brain. Second, since the neutral form of the chromophore is not absorbing the 470 nm light, the Ca^2+^ sensor is only susceptible to bleaching during the brief periods that the cell is active. This is also true for two-photon excitation with 930 nm light. The Ca^2+^ dependent equilibrium of the GCaMP6m chromophore essentially helps the Ca^2+^ sensor "hide in the dark" when there is no activity to report, making these biosensors uniquely resistant to photobleaching. This will be a beneficial property to incorporate into any future biosensor designs, especially for applications involving high frame rates and increased excitation light intensity.

## Materials and methods

### Imaging intracellular Ca^2+^ response in HEK293 cells

HEK293 cells were maintained in Dulbecco's Modified Eagle Medium, High Glucose (DMEM) and supplemented with 8% fetal bovine serum (FBS). Cells were plated in 24-well glass bottom plates coated with poly-D-lysine and kept in an incubator at 37°C with 5% CO_2_. Transient transfection were done with lipofectamine following the manufacturer's protocol (Invitrogen). Cells were co-transfected with pGP-CMV-GCaMP6m (Addgene, Plasmid #40754) and an M1 muscarinic receptor in the CMV expression vector pUB2.1 (Addgene, Plasmid #40728). For imaging, DMEM was replaced with warmed Dulbecco's Phosphate Buffered Saline (DPBS) with Ca^2+^ and Mg^2+^. The wide-field imaging experiments were performed on an Olympus IX70 microscope fitted with Sutter filter wheels and a 20x dry lens, alternating excitation with 410/20 nm and 480/20 nm bandpass filters and collecting emission with a 515 nm long-pass filter and CCD camera (Hamamatsu). To elicit intracellular Ca^2+^ release, carbachol was added to the well to a final concentration of 50 μM.

### Preparation of purified protein samples

The coding region of GCaMP6m was moved into a constitutive bacterial expression vector (pCP, generous gift from Nathan Shaner), using ligation-independent cloning [49]. E. coli colonies expressing GCaMP6m were picked for presence of 470 nm excited fluorescence and grown at 34°C for 48 hours in Circlegrow (MP Biomedicals) with Ampicillin. Bacterial pellets were lysed using BugBuster (Novagen) and Benzonase (Novagen). Cleared lysates were then His-tag purified using Protino Ni-TED 2000 packed columns (Macherey-Nagel). Purified fluorescent proteins were eluted in 1x PBS with imidazole pH 8 buffer solution. The protein was then concentrated and buffer exchanged into a pH 7.2 MOPS buffer using Vivaspin Turbo 15 (Sartorius) polyethersulfone ultrafiltration columns. The same buffer exchange columns were used to make all Ca^2+^-free and Ca^2+^-saturated samples for protein measurements, except for the Ca^2+^ titration experiments. All purified GCaMP6m Ca^2+^-free protein measurements for this work were carried out in 10 mM EGTA /100 mM KCl / 30 mM MOPS at pH 7.2. All purified GCaMP6m Ca^2+^-saturated protein measurements for this work were carried out in 10 mM CaEGTA /100 mM KCl /30 mM MOPS at pH 7.2.

### Ca^2+^ titration measurements

Ca^2+^ titration experiments for purified protein samples were done using Ca^2+^ Calibration Buffer Kit #1 (Life Technologies), following their protocol. We measured fluorescence intensity at the spectral peak (between 512 nm and 516 nm) as a function of Ca^2+^ concentration by varying CaEGTA concentrations of the 2 mL sample (0 to 10 mM CaEGTA). We used excitation at 470 nm to measure the Ca^2+^ dependent fluorescence of the anionic form, and 400 nm for the neutral form. The pH of the sample was measured at beginning and end of each titration series to ensure there was no shift in pH during titration. For the apparent K_d_ and Hill coefficient values from the 470 nm excited measurements, we averaged 3 separate Ca^2+^ titration measurements.

### Fluorescence spectra, fluorescence excitation spectra, and quantum yield measurements

Fluorescence spectra and fluorescence excitation spectra of purified protein samples were collected using a PC1™ spectrofluorimeter (ISS). Fluorescence spectra were corrected for the detection spectral sensitivity using the quinine sulfate solution in 1M H_2_SO_4_, whose corrected spectrum is published [65]. Fluorescence excitation spectra were corrected for excitation spectral variations using the styryl 9M (Aldrich) solution in ethanol, by comparing its excitation spectrum with its independently measured absorption spectrum. Fluorescence quantum yield (φ) of the GCaMP6m anionic chromophore (fluorescence peak for Ca^2+^-saturated at 513 nm; for Ca^2+^-free at 516 nm) upon its direct excitation at 470 nm was measured versus fluorescein in 1M NaOH (φ = 0.95) as a reference standard [65]. Fluorescence quantum yield (φ) of the GCaMP6m neutral chromophore (fluorescence peak for Ca^2+^-saturated at 512 nm, for Ca^2+^-free at 514 nm) was measured using excitation at 380 nm versus quinine sulfate in 1M H_2_SO_4_ (φ = 0.53) as a reference standard [65]. The fluorescence spectrum excited at 380 nm contains a minor short-wavelength contribution due to the direct emission from neutral chromophore near 450–470 nm. To get rid of this contribution, we used the fluorescence spectrum of Ca^2+^-free form excited at 470 nm (which lacks this contribution) and normalized it to the maximum of the spectrum excited at 380 nm (see S3 Fig). This normalized spectrum was then integrated. The optical density of the solutions at each excitation wavelength and in the whole range of fluorescence was kept less than 0.07. Corrected integrated spectra were used to calculate quantum yields [65].

### Extinction coefficient measurements

To measure extinction coefficients of the neutral and anionic forms of the chromophore in GCaMP6m (either in Ca^2+^-free or Ca^2+^-saturated states) separately, we measured the changes of the corresponding absorption spectrum upon alkaline pH titration of the purified protein solutions (cf. [66]). Absorption measurements were performed with a BioMate™ S3 spectrophotometer (ThermoFisher). To gradually change pH from 7.2 to >12, small volumes of 1M NaOH were added (1–5 μL) to the 2 mL sample. The pH of the sample was directly measured for each step, using an Orion™ PeropHecT™ ROSS™ combination pH microelectrode (ThermoFisher). In this titration, if the neutral form interconverts to the anionic form (i.e. before the third, denatured form appears), then at each step of the titration the changes of the concentration of the anionic and neutral forms, n_A_ and n_N_, are directly related:

$$\Delta n_{A}=-\Delta n_{N}$$ (6)

The changes in optical densities of the anionic and neutral forms at each stage of titration are:

$$\Delta {OD}_{A}=\varepsilon_{A}∙\Delta n_{A}$$ (7)

and,

$$\Delta {OD}_{N}=\varepsilon_{N}∙\Delta n_{N}$$ (8)

Therefore, the dependence of OD_A_ on OD_N_ (in the region of pH where only two forms are present) will give the straight line with a slope equal to:

$$\frac{\Delta {OD}_{A}}{{\Delta OD}_{N}}=\frac{{-\varepsilon }_{A}}{\varepsilon_{N}}$$ (9)

An additional equation involving ε_A_ and ε_N_ is obtained from the fact that the total concentration of chromophore before the titration (index 0) n^(0)^ = n^(0)^_A_ + n^(0)^_N_ is equal to the concentration of chromophore in alkaline denatured form n^(f)^_D_ (where index f corresponds to the final stage of denaturation before the onset of chemical degradation of the chromophore) which absorbs at 447 nm and has an extinction coefficient of 44,100 M^-1^ cm^-1^ [29]. Therefore:

$$n_{D}^{\left(f\right)}=n_{A}^{\left(0\right)}+n_{N}^{\left(0\right)}$$ (10)

or,

$$\frac{{OD}^{\left(f\right)}(447\;nm)}{44,100}=\frac{{OD}_{A}^{\left(0\right)}}{\varepsilon_{A}}+\frac{{OD}_{N}^{\left(0\right)}}{\varepsilon_{N}}$$ (11)

In this equation, everything is known except ε_A_ and ε_N_. Combining Eqs (9) and (11) gives us ε_A_ and ε_N_.

### pK_a_ measurements

We independently measured the changes in fluorescence spectra for alkaline and acidic titration experiments with purified protein samples. To do this we used the same alkaline titration method as the extinction coefficient measurements, and for acidic titration method we gradually added small volumes of 1M HCl (1–3 μl) to the 2 mL sample, from pH 7.2 to 4.3. pH (measured with Orion™ PeropHecT™ ROSS™ combination pH microelectrode, ThermoFisher) and corresponding fluorescence spectra (measured with spectrofluorimeter, PC1 ISS) were collected upon excitation of anionic form at 470 nm for the Ca^2+^-saturated and Ca^2+^-free states. To calculate the pK_a_, the peak fluorescence intensity near 515 nm was plotted against pH to get a pH titration curve. The data were then fit using two-binding site model function in the range of pH 4.3 to 11.4 for Ca^2+^-free GCaMP6m, and using a single-binding site model function for Ca^2+^-saturated GCaMP6m in the range of pH 4.8 to 8.3.

### Two-photon measurements

Ca^2+^-free and Ca^2+^-saturated purified GCaMP6m protein samples (5 x 10^−5^ M) were measured in 0.1 cm glass spectroscopy cuvettes (Starna cells). Two-photon excitation (TPE) spectra were measured using an MOM Sutter Instrument two-photon fluorescent microscope coupled with an Insight DeepSee (Newport) femtosecond laser tunable from 680 to 1300 nm in a similar approach as described in [67]. A Plan NeoFluar 2.5x/0.075 Zeiss objective was used to excite and collect fluorescence, which was passed through a Chroma ET 520/40m filter before reaching the PMT. To correct the TPE spectra for the wavelength-to-wavelength variations of laser properties (pulse duration and beam shape), fluorescein in a NaOH solution (pH 11) was used as a reference standard [68,69]. The TPE fluorescence had quadratic dependence on excitation power in the whole spectral range presented in our results section. Two-photon cross-section was measured at 790 nm for the neutral form of the chromophore in Ca^2+^-free GCaMP6m and 930 nm for the anionic form in the Ca^2+^-saturated and Ca^2+^-free GCaMP6m, using coumarin 485 in methanol (for 790 nm excitation) and fluorescein in NaOH aqueous solution (pH 11) (for 930 nm excitation) as reference standards [69]. Fluorescence intensity, *F*, as a function of excitation power, *P*, was measured for both the sample and the reference in the same conditions through a 507/10 nm narrow-bandpass filter. From the fit of these dependences to a quadratic function, *F = α P*^*2*^, factors *α* values were obtained and then normalized to the concentrations (obtained spectrophotometrically, BioMate™ S3 spectrophotometer) and differential quantum efficiencies at 507 nm (obtained with spectrofluorimeter, PC1 ISS).

## Supporting information

S1 Fig Emission spectra for purified GCaMP6m protein. A and B) Emission spectra for purified GCaMP6m protein in the Ca^2+^-free (blue, right axis) and Ca^2+^-saturated (black, left axis) states, excited at 400 nm (A) and 470 nm (B). Inset graphs: zoomed in view of Ca^2+^-dependent peak emission shift.
(TIF)

S2 Fig There is an optimal excitation wavelength to measure ΔF/F_0_ for GCaMP6m. Excitation spectra for the anionic chromophore using purified GCaMP6m protein in the Ca^2+^-free (dotted black trace) and Ca^2+^-saturated (solid black trace) states, fluorescence emission of the anionic form collected at 550 nm. The ratio of the Ca^2+^-saturated and Ca^2+^-free spectra illustrates the ΔF/F_0_ wavelength dependence (red trace).
(TIF)

S3 Fig Emission spectra used to resolve the fluorescence quantum yield for the Ca^2+^-free neutral form emitting near 515 nm. Fluorescence spectra of Ca^2+^-free GCaMP6m excited at 380 nm (black trace) and at 470 nm (red trace). The 470 nm excited spectrum was normalized to the maximum fluorescence intensity of the 380 nm excited spectrum.
(TIF)

S4 Fig Two-photon absorption spectra for Ca^2+^-saturated GCaMP6m (black solid circles, left x-axis and bottom y-axis). The cross-section of the Ca^2+^-saturated anionic chromophore, 50 GM, is the absolute cross-section at 930 nm excitation. Light grey arrow (B) marks the weak shoulder at ~990 nm that corresponds to the pure electronic transition of the anionic form, which occurs at double the wavelength of the one-photon peak absorption. B) Two-photon excitation spectra for Ca^2+^-free GCaMP6m (black open circles, left x-axis and bottom y-axis). The cross-section of the Ca^2+^-free neutral chromophore, 140 GM, is the absolute cross-section at 790 nm excitation, and the cross-section of the Ca^2+^-free anionic chromophore, 54 GM, is the absolute cross-section at 930 nm excitation. For both Ca^2+^-saturated (A) and Ca^2+^-free (B), the one-photon absorption is illustrated by the red solid trace (right x-axis, top y-axis).
(TIF)

## References

[1] Chen T-W, Wardill TJ, Sun Y, Pulver SR, Renninger SL, Baohan A, et al Ultrasensitive fluorescent proteins for imaging neuronal activity. Nature. 2013;499: 295–300. 10.1038/nature12354 23868258 PMC3777791

[2] Boyd AM, Kato HK, Komiyama T, Isaacson JS. Broadcasting of cortical activity to the olfactory bulb. Cell Rep. 2015;10: 1032–1039. 10.1016/j.celrep.2015.01.047 25704808 PMC4342299

[3] Dana H, Chen T-W, Hu A, Shields BC, Guo C, Looger LL, et al Thy1-GCaMP6 transgenic mice for neuronal population imaging in vivo. PLoS One. 2014;9: e108697 10.1371/journal.pone.0108697 25250714 PMC4177405

[4] Hinckley CA, Alaynick WA, Gallarda BW, Hayashi M, Hilde KL, Driscoll SP, et al Spinal Locomotor Circuits Develop Using Hierarchical Rules Based on Motorneuron Position and Identity. Neuron. 2015;87: 1008–1021. 10.1016/j.neuron.2015.08.005 26335645 PMC4592696

[5] Theis L, Berens P, Froudarakis E, Reimer J, Román Rosón M, Baden T, et al Benchmarking Spike Rate Inference in Population Calcium Imaging. Neuron. 2016;90: 471–482. 10.1016/j.neuron.2016.04.014 27151639 PMC4888799

[6] Grover D, Katsuki T, Greenspan RJ. Flyception: imaging brain activity in freely walking fruit flies. Nat Methods. 2016;13: 569–572. 10.1038/nmeth.3866 27183441

[7] Heckscher ES, Zarin AA, Faumont S, Clark MQ, Manning L, Fushiki A, et al Even-Skipped+ Interneurons Are Core Components of a Sensorimotor Circuit that Maintains Left-Right Symmetric Muscle Contraction Amplitude. Neuron. 2015;88: 314–329. 10.1016/j.neuron.2015.09.009 26439528 PMC4619170

[8] Baird GS, Zacharias DA, Tsien RY. Circular permutation and receptor insertion within green fluorescent proteins. Proc Natl Acad Sci U S A. 1999;96: 11241–11246. 10500161 10.1073/pnas.96.20.11241 PMC18018

[9] Ding J, Luo AF, Hu L, Wang D, Shao F. Structural basis of the ultrasensitive calcium indicator GCaMP6. Sci China Life Sci. 2014;57: 269–274. 10.1007/s11427-013-4599-5 24390420

[10] Tian L, Hires SA, Mao T, Huber D, Chiappe ME, Chalasani SH, et al Imaging neural activity in worms, flies and mice with improved GCaMP calcium indicators. Nat Methods. 2009;6: 875–881. 10.1038/nmeth.1398 19898485 PMC2858873

[11] Akerboom J, Rivera JDV, Guilbe MMR, Malave ECA, Hernandez HH, Tian L, et al Crystal Structures of the GCaMP Calcium Sensor Reveal the Mechanism of Fluorescence Signal Change and Aid Rational Design. J Biol Chem. 2008;284: 6455–6464. 10.1074/jbc.M807657200 19098007 PMC2649101

[12] Nagai T, Sawano A, Park ES, Miyawaki A. Circularly permuted green fluorescent proteins engineered to sense Ca2+. Proc Natl Acad Sci U S A. 2001;98: 3197–3202. 10.1073/pnas.051636098 11248055 PMC30630

[13] Zhao Y, Araki S, Wu J, Teramoto T, Chang YF, Nakano M, et al An Expanded Palette of Genetically Encoded Ca2+ Indicators. Science. 2011;333: 1888–1891. 10.1126/science.1208592 21903779 PMC3560286

[14] Akerboom J, Chen T-W, Wardill TJ, Tian L, Marvin JS, Mutlu S, et al Optimization of a GCaMP calcium indicator for neural activity imaging. J Neurosci. 2012;32: 13819–13840. 10.1523/JNEUROSCI.2601-12.2012 23035093 PMC3482105

[15] Wu J, Abdelfattah AS, Miraucourt LS, Kutsarova E, Ruangkittisakul A, Zhou H, et al A long Stokes shift red fluorescent Ca2+ indicator protein for two-photon and ratiometric imaging. Nat Commun. 2014;5: 5262–5211. 10.1038/ncomms6262 25358432 PMC4920544

[16] Ohkura M, Matsuzaki M, Kasai H, Imoto K, Nakai J. Genetically encoded bright Ca2+ probe applicable for dynamic Ca2+ imaging of dendritic spines. Anal Chem. 2005;77: 5861–5869. 10.1021/ac0506837 16159115

[17] Nakai J, Ohkura M, Imoto K. A high signal-to-noise Ca(2+) probe composed of a single green fluorescent protein. Nat Biotechnol. 2001;19: 137–141. 10.1038/84397 11175727

[18] Wang Q, Shui B, Kotlikoff MI, Sondermann H. Structural basis for calcium sensing by GCaMP2. Structure. 2008;16: 1817–1827. 10.1016/j.str.2008.10.008 19081058 PMC2614139

[19] Muto A, Ohkura M, Kotani T, Higashijima S-I, Nakai J, Kawakami K. Genetic visualization with an improved GCaMP calcium indicator reveals spatiotemporal activation of the spinal motor neurons in zebrafish. Proc Natl Acad Sci U S A. 2011;108: 5425–5430. 10.1073/pnas.1000887108 21383146 PMC3069178

[20] Cui G, Jun SB, Jin X, Pham MD, Vogel SS, Lovinger DM, et al Concurrent activation of striatal direct and indirect pathways during action initiation. Nature. 2013;494: 238–242. 10.1038/nature11846 23354054 PMC4039389

[21] Warp E, Agarwal G, Wyart C, Friedmann D, Oldfield CS, Conner A, et al Emergence of patterned activity in the developing zebrafish spinal cord. Curr Biol. 2012;22: 93–102. 10.1016/j.cub.2011.12.002 22197243 PMC3267884

[22] Chen Q, Cichon J, Wang W, Qiu L, Lee S-JR, Campbell NR, et al Imaging neural activity using Thy1-GCaMP transgenic mice. Neuron. 2012;76: 297–308. 10.1016/j.neuron.2012.07.011 23083733 PMC4059513

[23] Drobizhev M, Callis PR, Nifosì R, Wicks G, Stoltzfus C, Barnett L, et al Long- and Short-Range Electrostatic Fields in GFP Mutants: Implications for Spectral Tuning. Sci Rep. 2015;5: 13223 10.1038/srep13223 26286372 PMC4541067

[24] Jung G, Wiehler J, Zumbusch A. The photophysics of green fluorescent protein: influence of the key amino acids at positions 65, 203, and 222. Biophys J. 2005;88: 1932–1947. 10.1529/biophysj.104.044412 15613627 PMC1305246

[25] Lossau H, Kummer A, Heinecke R, Pöllinger-Dammer F, Kompa C, Bieser G, et al Time-resolved spectroscopy of wild-type and mutant Green Fluorescent Proteins reveals excited state deprotonation consistent with fluorophore-protein interactions. Chem Phys. 1996;213: 1–16.

[26] Chattoraj M, King BA, Bublitz GU, Boxer SG. Ultra-fast excited state dynamics in green fluorescent protein: multiple states and proton transfer. Proc Natl Acad Sci U S A. 1996;93: 8362–8367. 8710876 10.1073/pnas.93.16.8362 PMC38676

[27] Brejc K, Sixma TK, Kitts PA, Kain SR, Tsien RY, Ormö M, et al Structural basis for dual excitation and photoisomerization of the Aequorea victoria green fluorescent protein. Proc Natl Acad Sci U S A. 1997;94: 2306–2311. 9122190 10.1073/pnas.94.6.2306 PMC20083

[28] Tallini YN, Ohkura M, Choi B-R, Ji G, Imoto K, Doran R, et al Imaging cellular signals in the heart in vivo: Cardiac expression of the high-signal Ca2+ indicator GCaMP2. Proc Natl Acad Sci U S A. 2006;103: 4753–4758. 10.1073/pnas.0509378103 16537386 PMC1450242

[29] Ward WW. Biochemical and Physical Properties of Green Fluorescent Protein In: Chalfie M, Kain SR, editors. Green Fluorescent Protein. Hoboken, NJ, USA: John Wiley & Sons; 2005 pp. 39–65.

[30] Shu X, Shaner NC, Yarbrough CA, Tsien RY, Remington SJ. Novel chromophores and buried charges control color in mFruits. Biochemistry. 2006;45: 9639–9647. 10.1021/bi060773l 16893165

[31] Warshel A. Calculations of enzymic reactions: calculations of pKa, proton transfer reactions, and general acid catalysis reactions in enzymes. Biochemistry. 1981;20: 3167–3177. 7248277 10.1021/bi00514a028

[32] Martin ME, Negri F, Olivucci M. Origin, Nature, and Fate of the Fluorescent State of the Green Fluorescent Protein Chromophore at the CASPT2//CASSCF Resolution. J Am Chem Soc. 2004;126: 5452–5464. 10.1021/ja037278m 15113217

[33] Zhang M-Y, Xu C, Lin C-S, Guan X, Cheng W-D. Theoretical study of the proton transfer wires influence on the one- and two-photon absorption properties of green fluorescent protein chromophore. Org Biomol Chem. 2013;11: 1414–1422. 10.1039/c2ob26914g 23338242

[34] Kuboniwa H, Tjandra N, Grzesiek S, Ren H, Claude B, Bax A. Solution structure of calcium-free calmodulin. Nat Struct Biol. 1995;2 Available: http://spin.niddk.nih.gov/bax/lit/spdf/225.pdf 10.1038/nsb0995-768 7552748

[35] Chen Y, Song X, Ye S, Miao L, Zhu Y, Zhang R-G, et al Structural insight into enhanced calcium indicator GCaMP3 and GCaMPJ to promote further improvement. Protein Cell. 2013;4: 299–309. 10.1007/s13238-013-2103-4 23549615 PMC4875517

[36] Xie D, Zeng J. Electronic excitations of green fluorescent proteins: protonation states of chromophore model compound in solutions. J Comput Chem. 2005;26: 1487–1496. 10.1002/jcc.20273 16092146

[37] Scott JN, Callis PR. Insensitivity of tryptophan fluorescence to local charge mutations. J Phys Chem B. 2013;117: 9598–9605. 10.1021/jp4041716 23883101

[38] Drobizhev M, Makarov NS, Tillo SE, Hughes TE, Rebane A. Two-photon absorption properties of fluorescent proteins. Nat Methods. 2011;8: 393–399. 10.1038/nmeth.1596 21527931 PMC4772972

[39] Sarkisyan KS, Goryashchenko AS, Lidsky PV, Gorbachev DA, Bozhanova NG, Gorokhovatsky AY, et al Green fluorescent protein with anionic tryptophan-based chromophore and long fluorescence lifetime. Biophys J. 2015;109: 380–389. 10.1016/j.bpj.2015.06.018 26200874 PMC4621817

[40] Drobizhev M, Makarov NS, Tillo SE, Hughes TE, Rebane A. Describing two-photon absorptivity of fluorescent proteins with a new vibronic coupling mechanism. J Phys Chem B. 2012;116: 1736–1744. 10.1021/jp211020k 22224830 PMC3280616

[41] Stoltzfus CR, Barnett LM, Drobizhev M, Wicks G, Mikhaylov A, Hughes TE, et al Two-photon directed evolution of green fluorescent proteins. Sci Rep. 2015;5: 11968 10.1038/srep11968 26145791 PMC4491718

[42] Akerboom J, Carreras Calderón N, Tian L, Wabnig S, Prigge M, Tolö J, et al Genetically encoded calcium indicators for multi-color neural activity imaging and combination with optogenetics. Front Mol Neurosci. 2013;6: 1–29. 10.3389/fnmol.2013.00002 PMC3586699 23459413

[43] Bhargava Y, Hampden-Smith K, Chachlaki K, Wood KC, Vernon J, Allerston CK, et al Improved genetically-encoded, FlincG-type fluorescent biosensors for neural cGMP imaging. Front Mol Neurosci. 2013;6: 26 10.3389/fnmol.2013.00026 24068983 PMC3781335

[44] Odaka H, Arai S, Inoue T, Kitaguchi T. Genetically-Encoded Yellow Fluorescent cAMP Indicator with an Expanded Dynamic Range for Dual-Color Imaging. PLoS One. 2014;9: e100252 10.1371/journal.pone.0100252 24959857 PMC4069001

[45] Hung YP, Albeck JG, Tantama M, Yellen G. Imaging Cytosolic NADH-NAD+ Redox State with a Genetically Encoded Fluorescent Biosensor. Cell Metab. 2011;14: 545–554. 10.1016/j.cmet.2011.08.012 21982714 PMC3190165

[46] Marvin JS, Schreiter ER, Echevarría IM, Looger LL. A genetically encoded, high-signal-to-noise maltose sensor. Proteins: Struct Funct Bioinf. 2011;79: 3025–3036. 10.1002/prot.23118 PMC3265398 21989929

[47] Shen Y, Rosendale M, Campbell RE, Perrais D. pHuji, a pH-sensitive red fluorescent protein for imaging of exo-and endocytosis. J Cell Biol. 2014;207: 419–432. 10.1083/jcb.201404107 25385186 PMC4226733

[48] Marvin JS, Borghuis BG, Tian L, Cichon J, Harnett MT, Akerboom J, et al An optimized fluorescent probe for visualizing glutamate neurotransmission. Nat Methods. 2013;10: 162–170. 10.1038/nmeth.2333 23314171 PMC4469972

[49] Barnett L, Platisa J, Popovic M, Pieribone VA, Hughes T. A fluorescent, genetically-encoded voltage probe capable of resolving action potentials. PLoS One. 2012;7: e43454 10.1371/journal.pone.0043454 22970127 PMC3435330

[50] Nikolaev VO, Gambaryan S, Lohse MJ. Fluorescent sensors for rapid monitoring of intracellular cGMP. Nat Methods. 2006;3: 23–25. 10.1038/nmeth816 16369548

[51] St-Pierre F, Marshall JD, Yang Y, Gong Y, Schnitzer MJ, Lin MZ. High-fidelity optical reporting of neuronal electrical activity with an ultrafast fluorescent voltage sensor. Nat Neurosci. 2014;17: 884–889. 10.1038/nn.3709 24755780 PMC4494739

[52] De Michele R, Ast C, Loqué D, Ho C-H, Andrade SLA, Lanquar V, et al Fluorescent sensors reporting the activity of ammonium transceptors in live cells. Elife. 2013;2: e00800 10.7554/eLife.00800 23840931 PMC3699834

[53] Ast C, De Michele R, Kumke MU, Frommer WB. Single-fluorophore membrane transport activity sensors with dual-emission read-out. Elife. 2015;4: e07113 10.7554/eLife.07113 26090909 PMC4491562

[54] Belousov VV, Fradkov AF, Lukyanov KA, Staroverov DB, Shakhbazov KS, Terskikh AV, et al Genetically encoded fluorescent indicator for intracellular hydrogen peroxide. Nat Methods. 2006;3: 281–286. 10.1038/nmeth866 16554833

[55] Berg J, Hung YP, Yellen G. A genetically encoded fluorescent reporter of ATP:ADP ratio. Nat Methods. 2009;6: 161–166. 10.1038/nmeth.1288 19122669 PMC2633436

[56] Inoue M, Takeuchi A, Horigane S, Ohkura M. Rational design of a high-affinity, fast, red calcium indicator R-CaMP2. Nature. 2015; Available: http://www.nature.com/nmeth/journal/v12/n1/abs/nmeth.3185.html 10.1038/nmeth.3185 25419959

[57] Tewson P, Westenberg M, Zhao Y, Campbell RE, Quinn AM, Hughes TE. Simultaneous detection of Ca 2+ and diacylglycerol signaling in living cells. PLoS One. 2012;7: e42791 10.1371/journal.pone.0042791 22912738 PMC3422227

[58] Jin L, Han Z, Platisa J, Wooltorton JRA, Cohen LB, Pieribone VA. Single action potentials and subthreshold electrical events imaged in neurons with a fluorescent protein voltage probe. Neuron. 2012;75: 779–785. 10.1016/j.neuron.2012.06.040 22958819 PMC3439164

[59] Kang BE, Baker BJ. Pado, a fluorescent protein with proton channel activity can optically monitor membrane potential, intracellular pH, and map gap junctions. Sci Rep. 2016;6: 23865 10.1038/srep23865 27040905 PMC4878010

[60] Miesenböck G, De Angelis DA, Rothman JE. Visualizing secretion and synaptic transmission with pH-sensitive green fluorescent proteins. Nature. 1998;394: 192–195. 10.1038/28190 9671304

[61] Sankaranarayanan S, De Angelis D, Rothman JE, Ryan TA. The use of pHluorins for optical measurements of presynaptic activity. Biophys J. 2000;79: 2199–2208. 10.1016/S0006-3495(00)76468-X 11023924 PMC1301110

[62] Han Z, Jin L, Chen F, Loturco JJ, Cohen LB, Bondar A, et al Mechanistic studies of the genetically encoded fluorescent protein voltage probe ArcLight. PLoS One. 2014;9: e113873 10.1371/journal.pone.0113873 25419571 PMC4242678

[63] Ataka K, Pieribone VA. A Genetically Targetable Fluorescent Probe of Channel Gating with Rapid Kinetics. Biophys J. 2002;82: 509–516. 10.1016/S0006-3495(02)75415-5 11751337 PMC1302490

[64] Treger JS, Priest MF, Bezanilla F. Single-molecule fluorimetry and gating currents inspire an improved optical voltage indicator. Elife. 2015; Available: http://elifesciences.org/content/4/e10482.abstract 10.7554/eLife.10482 PMC4658195 26599732

[65] Lakowicz JR. Principles of Fluorescence Spectroscopy. 3rd ed. New York: Springer; 2006.

[66] Hanson GT, McAnaney TB, Park ES, Rendell MEP, Yarbrough DK, Chu S, et al Green fluorescent protein variants as ratiometric dual emission pH sensors. 1. Structural characterization and preliminary application. Biochemistry. 2002;41: 15477–15488. 12501176 10.1021/bi026609p

[67] Tsai PS, Nishimura N, Yoder EJ, Dolnick EM, Allen White G, Kleinfeld D. Principles, Design, and Construction of a Two-Photon Laser-Scanning Microscope for In Vitro and In Vivo Brain Imaging In: Frostig RD, editor. In vivo optical imaging of brain function. Boca Raton: CRC Press; 2002 pp. 113–171.

[68] Xu C, Webb WW. Measurement of two-photon excitation cross sections of molecular fluorophores with data from 690 to 1050 nm. J Opt Soc Am B. 1996;13: 481–491.

[69] Makarov NS, Drobizhev M, Rebane A. Two-photon absorption standards in the 550–1600 nm excitation range: establishing a correction curve for accurate cross section calibration. Integrated Optoelectronic Devices 2008. International Society for Optics and Photonics; 2008. pp. 689105–689105–12.

