# Supplementary figures and images for: Deciphering the molecular mechanism responsible for GCaMP6m's Ca2+-dependent change in fluorescence
Source: PLoS One. 2017 Feb 9;12(2):e0170934. doi: 10.1371/journal.pone.0170934 (PMC5300113; doi:10.1371/journal.pone.0170934)

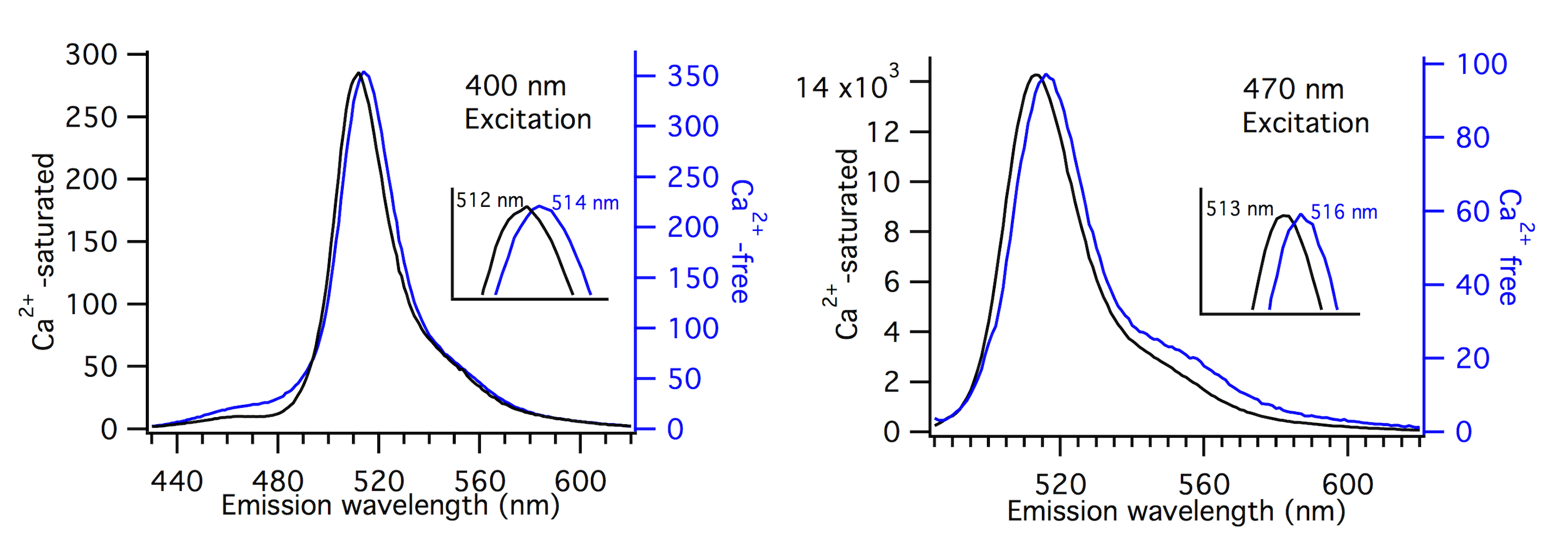

Supplement: S1 Fig — A and B) Emission spectra for purified GCaMP6m protein in the Ca2+-free (blue, right axis) and Ca2+-saturated (black, left axis) states, excited at 400 nm (A) and 470 nm (B). Inset graphs: zoomed in view of Ca2+-dependent peak emission shift. (TIF) [file pone.0170934.s001.tif]

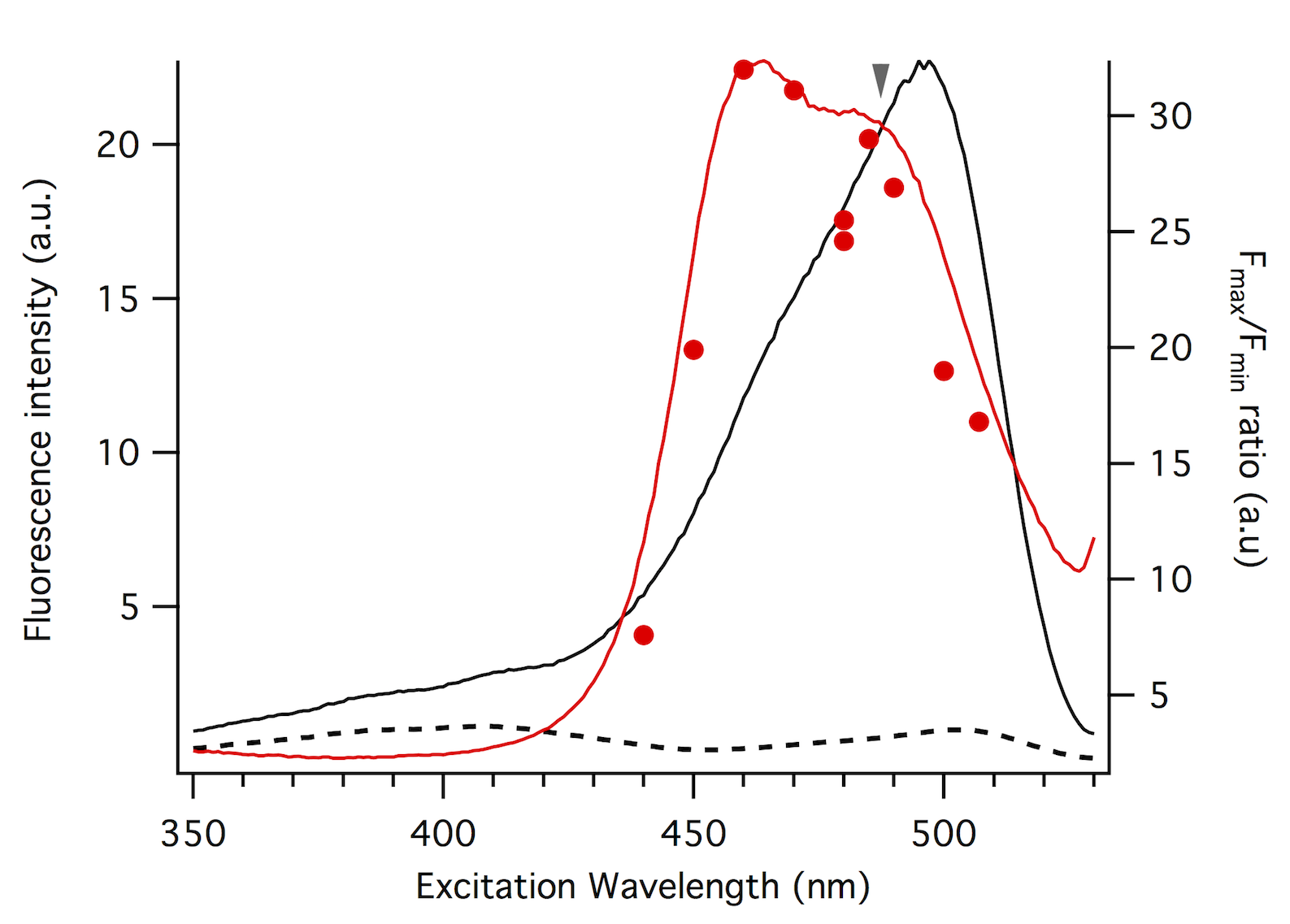

Supplement: S2 Fig — Excitation spectra for the anionic chromophore using purified GCaMP6m protein in the Ca2+-free (dotted black trace) and Ca2+-saturated (solid black trace) states, fluorescence emission of the anionic form collected at 550 nm. The ratio of the Ca2+-saturated and Ca2+-free spectra illustrates the ΔF/F0 wavelength dependence (red trace). (TIF) [file pone.0170934.s002.tif]

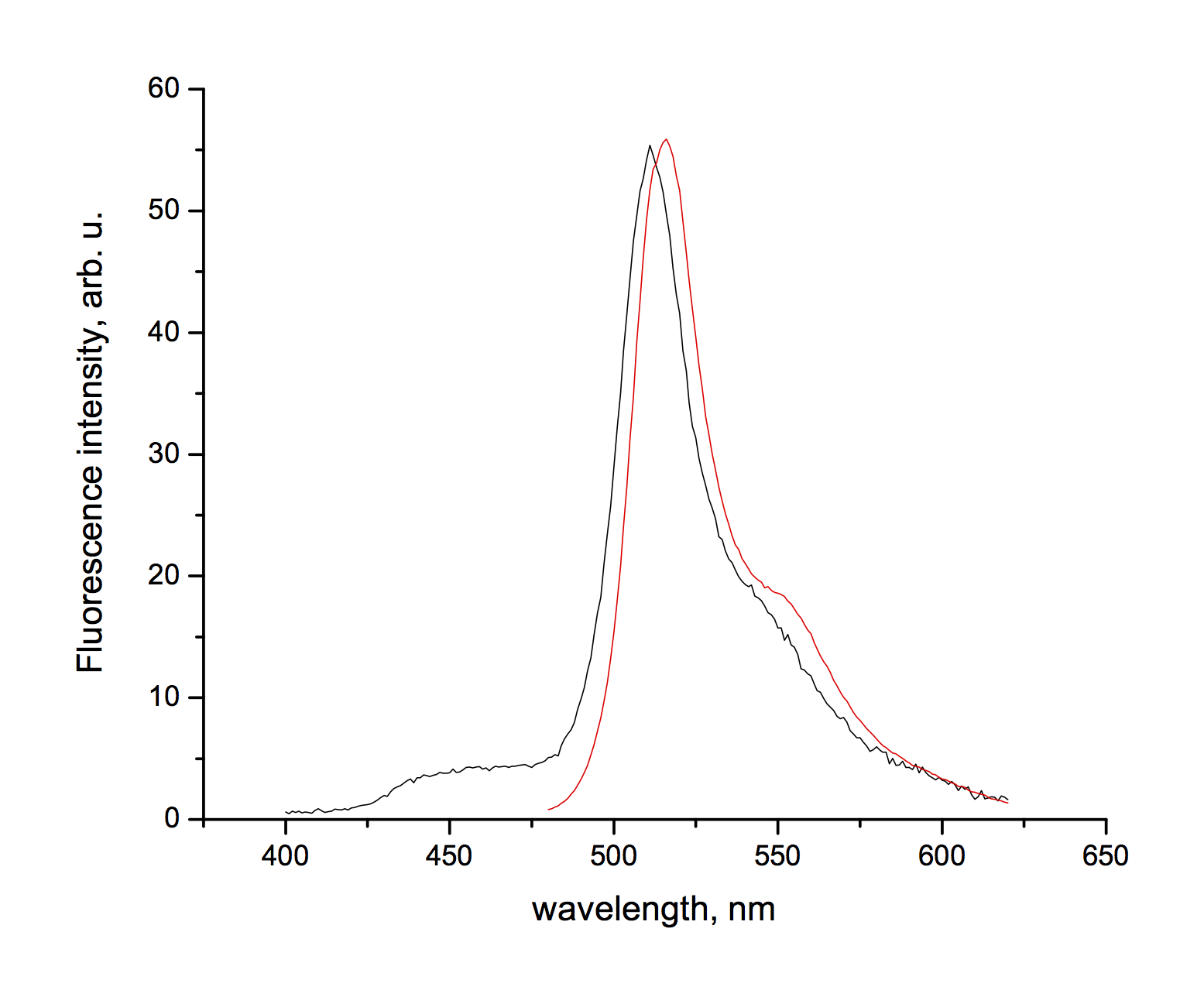

Supplement: S3 Fig — Fluorescence spectra of Ca2+-free GCaMP6m excited at 380 nm (black trace) and at 470 nm (red trace). The 470 nm excited spectrum was normalized to the maximum fluorescence intensity of the 380 nm excited spectrum. (TIF) [file pone.0170934.s003.tif]

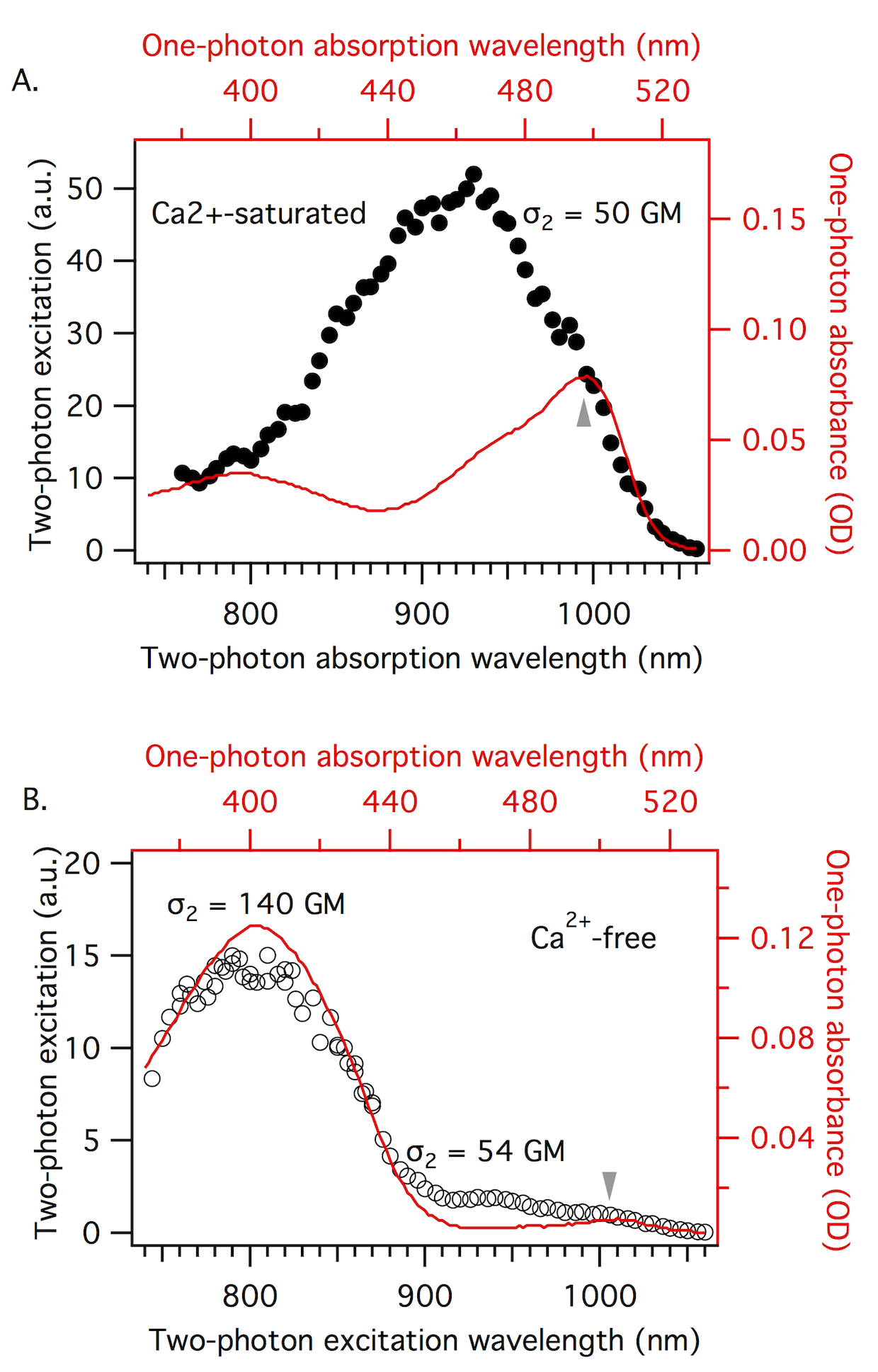

Supplement: S4 Fig — The cross-section of the Ca2+-saturated anionic chromophore, 50 GM, is the absolute cross-section at 930 nm excitation. Light grey arrow (B) marks the weak shoulder at ~990 nm that corresponds to the pure electronic transition of the anionic form, which occurs at double the wavelength of the one-photon peak absorption. B) Two-photon excitation spectra for Ca2+-free GCaMP6m (black open circles, left x-axis and bottom y-axis). The cross-section of the Ca2+-free neutral chromophore, 140 GM, is the absolute cross-section at 790 nm excitation, and the cross-section of the Ca2+-free anionic chromophore, 54 GM, is the absolute cross-section at 930 nm excitation. For both Ca2+-saturated (A) and Ca2+-free (B), the one-photon absorption is illustrated by the red solid trace (right x-axis, top y-axis). (TIF) [file pone.0170934.s004.tif]
